# Synthesis and Biological Evaluation of 1,2,3-Triazole Tethered Thymol-1,3,4-Oxadiazole Derivatives as Anticancer and Antimicrobial Agents

**DOI:** 10.3390/ph14090866

**Published:** 2021-08-28

**Authors:** Abdulraheem S. A. Almalki, Syed Nazreen, Azizah M. Malebari, Nada M. Ali, Ahmed A. Elhenawy, Abdullah A. A. Alghamdi, Abrar Ahmad, Sulaiman Y. M. Alfaifi, Meshari A. Alsharif, Mohammad Mahboob Alam

**Affiliations:** 1 Department of Chemistry, Faculty of Science, Taif University, Taif 21974, Saudi Arabia; almalki.a@tu.edu.sa; 2 Department of Chemistry, Faculty of Science, Albaha University, Albaha 65731, Saudi Arabia; syed.nazreen22885@gmail.com (S.N.); nada.m@bu.edu.sa (N.M.A.); ahmed.elhenawy@azhar.edu.eg (A.A.E.); 3 Department of Pharmaceutical Chemistry, Faculty of Pharmacy, King Abdulaziz University, Jeddah 21589, Saudi Arabia; amelibary@kau.edu.sa; 4 Chemistry Department, Faculty of Science, Al-Azhar University, Cairo 11884, Egypt; 5 Department of Biology, Faculty of Science, Albaha University, Albaha 65731, Saudi Arabia; aaa.alghamdi@bu.edu.sa; 6 Department of Biochemistry, Faculty of Science, King Abdulaziz University, Jeddah 21589, Saudi Arabia; abrar.ghauri@gmail.com; 7 Chemistry Department, Faculty of Science, King Abdulaziz University, Jeddah 21589, Saudi Arabia; Salfaifi@kau.edu.sa; 8 Chemistry Department, Faculty of Applied Sciences, Umm Al-Qura University, Makkah 21421, Saudi Arabia; Maasharif@uqu.edu.sa

**Keywords:** thymol, 1,2,3-triazole, 1,3,4-oxadiazole, cytotoxicity, antimicrobial, computational studies

## Abstract

A library of 1,2,3-triazole-incorporated thymol-1,3,4-oxadiazole derivatives (**6**–**18**) hasbeen synthesized and tested for anticancer and antimicrobial activities. Compounds **7**, **8**, **9**, **10**, and **11** exhibited significant antiproliferative activity. Among these active derivatives, compound 2-(4-((5-((2-isopropyl-5-methylphenoxy)methyl)-1,3,4-oxadiazol-2-ylthio)methyl)-1*H*-1,2,3-triazol-1-yl)phenol (**9**) was the best compound against all three tested cell lines, MCF-7 (IC_50_ 1.1 μM), HCT-116 (IC_50_ 2.6 μM), and HepG2 (IC_50_ 1.4 μM). Compound **9** was found to be better than the standard drugs, doxorubicin and 5-fluorouracil. These compounds showed anticancer activity through thymidylate synthase inhibition as they displayed significant TS inhibitory activity with IC_50_ in the range 1.95–4.24 μM, whereas the standard drug, Pemetrexed, showed IC_50_ 7.26 μM. The antimicrobial results showed that some of the compounds (**6**, **7**, **9**, **16**, and **17**) exhibited good inhibition on *Escherichia coli* (*E. coli*) and *Staphylococcus aureus* (*S. aureus*). The molecular docking and simulation studies supported the anticancer and antimicrobial data. It can be concluded that the synthesized 1,2,3-triazole tethered thymol-1,3,4-oxadiazole conjugates have both antiproliferative and antimicrobial potential.

## 1. Introduction

Cancer and infectious diseases are a major health burden on humankind globally [1]. According to the WHO, the mortality rates due to cancer will double in the coming years [2]. Chemotherapy is the mainstay for cancer treatment but suffers lack of selectivity, undesirable side effects, and multidrug resistance [3,4]. On the other hand, due to microbial resistance to the currently available antimicrobial drugs, infectious diseases also pose a serious problem to the medical community [5,6]. Cancer patients who have undergone chemotherapy treatments are more vulnerable to microbial infections due to impaired immunity [7]. Therefore, monotherapy with dual action as an anticancer and antimicrobial might be cost effective and reduce drug administration frequency, side effects, and antimicrobial resistance.

Heterocycles have played an important role in drug discovery. 1,2,3-triazole is a versatile scaffold that exhibits potential pharmacological activities such as anticancer, antimicrobial, anti-inflammatory, antitubercular, antiviral, and anti-HIV activities [8,9,10,11,12,13,14]. Drugs containing 1,2,3-triazole, tazobactam and cefatrizine, are used for antimicrobial and anticancer treatments. Additionally, 1,3,4-oxadiazole derivatives are multifunctional pharmacological pharmacophores which alter the physicochemical and pharmacokinetic properties of a drug. They are found in structures of many marketed drugs such as Raltegravir (antiviral), furamizole (antimicrobial), Zibotentan (anticancer), etc. [15]. It is very well known that natural products, due to their structural diversity and excellent pharmacological potential have provided numerous leads in drug discovery. Thymol, a monoterpenoid, is a major component of *Thymus vulgaris* L. which possesses anti-inflammatory, antimicrobial, antioxidant, and anticancer potential [16,17,18,19]. Recently, thymol-incorporated 1,2,3-triazole hybrids exerted significant anticancer activity [20].

**Figure 1:**
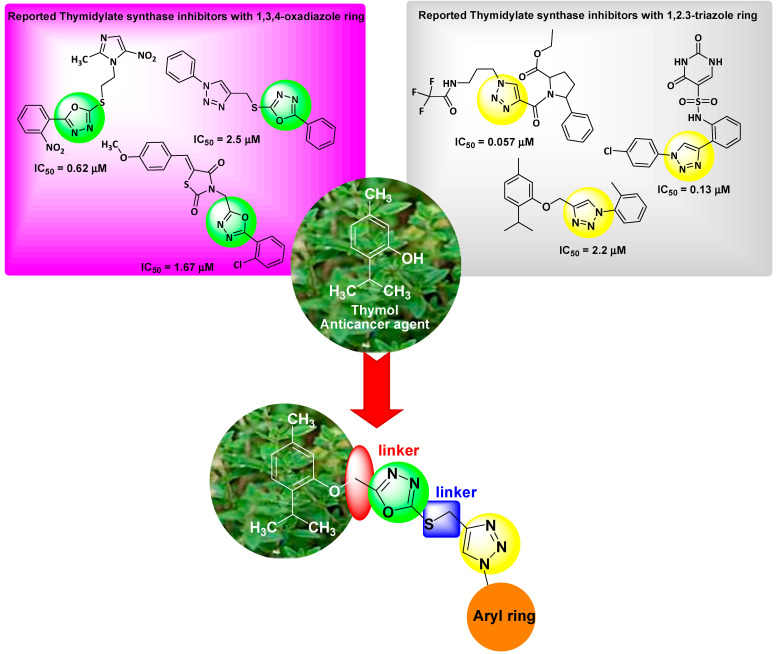
Rationale for the present work.

Thymidylate synthase inhibitors are evolving as a captivating target in chemotherapy [21]. These inhibitors induce apoptosis and cause cell cycle arrests by halting deoxythymidine monophosphate, which is responsible for formation of dTTP (a DNA synthesis precursor) [22]. From the literature, it is reported that 1,3,4-oxadiazole and 1,2,3-triazole derivatives exhibit antiproliferative activity by inhibiting thymidylate synthase [23,24,25,26,27,28] (Figure 1). Our research group has been involved in the development of anticancer leads targeting thymidylate synthase. In continuation of our previously reported work [20,23,26,29], we aim to incorporate both these heterocycles with thymol in a single molecule to generate new anticancer leads. Therefore, we report the synthesis of thymol-incorporated 1,3,4-oxadiazole and 1,2,3-triazole hybrids, anticancer and antimicrobial activities, as well as computational studies.

## 2. Results and Discussion

### 2.1. Chemistry

**Scheme 1:**
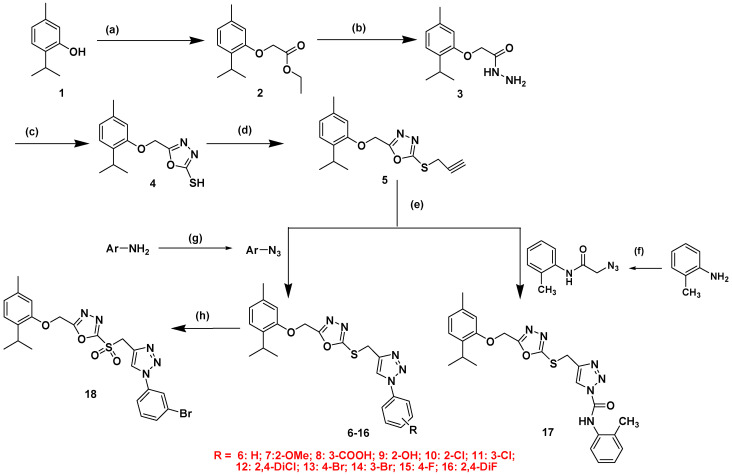
(**a**) K_2_CO_3_, ClCH_2_COOC_2_H_5_, reflux, 10 h; (**b**) MeOH, N_2_H_4_.H_2_O, reflux, 10 h; (**c**) i-Abs EtOH, KOH, CS_2_, stir, 24 h, ii- reflux, 10hrs; (**d**) acetone, K_2_CO_3_, Propargyl bromide; reflux, 5 h; (**e**) tert-BuOH:H_2_O (1:1), Na ascorbate, CuSO_4_·H_2_O, r.t., 5–11 h; (**f**) i. Dichloromethane (DCM), TEA, ClCH_2_COCl, stir, 0 to −5 °C; ii. NaN_3_, stir, 12 h; (**g**) HCl, NaNO_2_, NaN_3_, CH_3_COONa, stir, 0 to −5 °C, 1–2 h; (h) DCM, H_2_O_2_, stir, 0 to −5 °C, 28 h.

The route for the synthesis of final derivatives **6**–**18** is shown in Scheme 1. Compounds **6**–**17** were obtained by the Click chemistry approach using different aromatic azides and *S*-propargylated 1,3,4-oxadiazole intermediate **5**. Intermediate **5** was synthesized from thymol (**1**) by esterification, followed by treatment with hydrazinium hydroxide in absolute ethanol, which yielded intermediate **3** [30]. This intermediate **3,** on treatment with CS_2_ and KOH, afforded intermediate **4** which, upon propargylation using propargyl bromide in dry acetone, gave the key intermediate **5,** which was further used for the synthesis of final compounds **6**–**17**. Compound **18** was synthesized by the oxidation of compound **14** with hydrogen peroxide in dichloromethane. All the final compound structures were confirmed by different analytical techniques.

### 2.2. In Silico Absorption, Distribution, Metabolism and Elimination (ADME)/Pharmacokinetic Studies

In order to save cost and time in the drug development process, *in silico* studies are now used extensively in drug discovery. The desired pharmacokinetics are very important for a drug so as to avoid the problems of permeability, gastrointestinal absorption, solubility, transportation, and so on [31]. It is very well known that the orally available drug should follow the Lipinski rule of five; for example, it should have a molecular weight less than 500, and the hydrogen bond acceptor/donor should be less than 10 and 5, respectively. Furthermore, the lipophilicity of the drug should be less than five to avoid bioavailability problems. Therefore, all the final thymol hybrids were screened for *in silico* pharmacokinetics studies.

All the final compounds except **13**, **14,** and **18** exhibited a molecular weight less than 500, whereas the hydrogen bond acceptor and donor were found to be in an acceptable range for all the compounds, indicating that most of the compounds can be easily transported in the body. The highest % absorption of 73.07 was depicted by compounds **6**,**10**–**16** while other compounds showed % absorption in the range 60–70%. All the compounds displayed a desired TPSA in the range 104.16–141.46 and were non-permeable to the brain (Table 1).These data suggested that these compounds possess good pharmacokinetics and drug-likeness properties.

### 2.3. Biological Studies

#### 2.3.1. Antiproliferative Activity

The antiproliferative activity of the final derivatives **6**–**18** was performed on three cancer cell lines (MCF-7, HepG2 and HCT-116) by MTT assay [23]; 5-fluorouracil (5-FU) and doxorubicin were used as standard drugs (positive control). The antiproliferative activity depicted variable anticancer potential (Table 2). Two most promising compounds, **9** and **10** were found to be equipotent to doxorubicin (IC_50_ 1.2 μM) with IC_50_ 1.1 and 1.3 μM, respectively, whereas 17- and 15-times active to 5-florouracil (IC_50_ 18.74 μM) towards MCF-7 cells. Other compounds **7**, **8,** and **11** were more active than 5-FU towards the same cancer cells with IC_50_ in the range 2.4 μM–4.9 μM. Compound **17** (IC_50_ 9.39 μM) bearing the amide group was two-fold active to 5-FU, whereas sulfone-incorporated compound **18** was less active with IC_50_ 98.2 μM towards breast cancer cells, MCF-7.

Against HepG2 cells, compounds **7** and **9** were the most potent molecules with IC_50_ 1.8 μM and 1.4 μM, respectively. It was noted that compound **9** was better than doxorubicin (IC_50_ 1.8 μM) in exerting cytotoxicity, whereas **7** was equipotent to doxorubicin. Moreover, both the compounds were 20- and 16-fold active to 5-FU (IC_50_ 28.65 μM). Compounds **8** and **10** displayed better cytotoxicity than 5-FU with IC_50_ 5.3 μM and 2.5 μM, respectively. Other compounds, except **18,** showed moderate activity with IC_50_ in the range 18.6–69.4 μM. Towards HCT-116 cells, compound **9** was the best compound with IC_50_ 2.6 μM and equipotent to doxorubicin. 

Structure-activity relationships (SAR) deduced from the activity is as follows: (i) Position of the substituents: *Ortho* substituted compounds exhibited better activity than *meta* and *para*-substituted derivatives. Compound **7** (2-OMe), **9** (2-OH), and **10** (2-Cl) bearing *ortho* substitution on the phenyl ring were most active followed by compound **8** (3-COOH), **11** (3-Cl), and **13** (4-Br). (ii) Number of the substituents: Increase in the number of substituent decreased the activity: disubstituted derivatives **12** (2,4-diCl) and **16** (2,4-diF) diminished cytotoxicity compared with monosubstituted derivatives **10** and **11** (Cl) and **15** (F). (iii) Incorporation of the sulfone group in the structure displayed weak cytotoxicity.

#### 2.3.2. Thymidylate Synthase Activity

The potent derivatives from cytotoxicity results were selected for the TS enzymatic assay. Compounds **7**, **8**, **9**, **10,** and **11** and Pemetrexed (reference drug) were screened for TS inhibition. It was observed all these tested derivatives caused significant inhibition on the TS enzyme. Compound **9** having *o*-hydroxy was the best compound with IC_50_ 1.95 μM followed by compound **10** with IC_50_ 2.18 μM, whereas Pemetrexed inhibited TS with IC_50_ 7.26 μM. In terms of folds activity, compound **9** was 3.72-fold and **10** was 3.33-fold active to the reference drug, Pemetrexed. Other compounds **7**, **8,** and **11** were also better than Pemetrexed in TS inhibition with IC_50_ in the range 3.52–4.24 μM (Table 3). It was interesting to note that the incorporation of 1,3,4-oxadiazole and 1,2,3-triazole under one construct were better in TS inhibition [20]. These derivatives may be investigated further for development of anticancer leads.

#### 2.3.3. Antimicrobial Activity

The antimicrobial activity was performed on *E. coli*, *S. aureus* and *C. albicans*. It was found that compounds **5**, **6**, **7**, **9**, **12**, **13**, **14**, **16,** and **17** displayed zones of inhibition of 12 mm, 14 mm, 12 mm, 12 mm, 10 mm, 11 mm, 9 mm, 14 mm, and 12 mm, respectively, for *E. coli* (gram negative), whereas other compounds were not effective for *E. coli*. Gram-positive (*S. aureus*) compounds **5**, **6**, **7**, **8,9**, **16,** and **17** showed zones of inhibition of 9 mm, 10 mm, 12 mm, 10 mm, 12 mm, 15 mm, and 12 mm, respectively. Other samples did not display any zone of inhibition for *S. aureus*. The antifungal activity against *Candida albicans* was displayed by compounds **5**, **9,** and **12** as the formation of inhibition zones was found to be 10 mm, 10 mm, and 12 mm, respectively (Table 4). It was noticed that compounds **5**, **6**, **7**, **9**, **16**, and **17** had broad-spectrum antimicrobial potential for both gram-negative and positive microbial strains. These data indicated that derivatives **6** and **16** were most potent against *E. coli* with zones of inhibition of 14 mm each while compound **16** was most active against *S. aureus* with a zone of inhibition of 15 mm.

#### 2.3.4. Computational Studies

##### Electronic Properties

**Figure 2:**
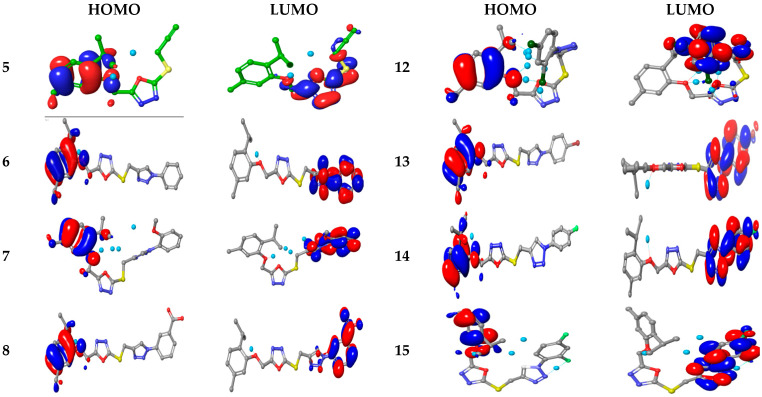

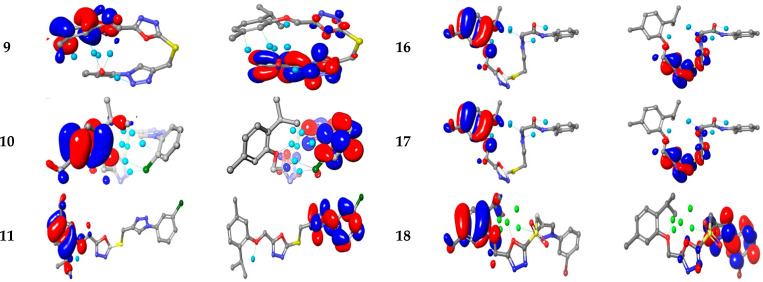
Ground state charge density of FMOs of thymol derivatives.

To understand the structural elements of thymol conjugates, frontier molecular orbitals energy are shown in Figure 2. The HOMO/LUMOs and HOMO–1/ LUMO+1 of thymol derivatives were probed at B3LYP/6-311+G(d,p) using the LANL2DZ level [32]. HOMO was observed over the thymol corner for compounds **5**–**18,** whereas the LUMO zone was compressed opposite phenyl corners for **5**–**15** and**18** but in compounds **16** and **17,** LUMO overlapped *bis*-triazole rings. This arrangement revealed that hybrids **5**–**17** possess intra-molecular charge transfer (ICT) between HOMOs to LUMOs. Compounds **5**–**18** correlated to the spatial distribution of molecular orbitals, enlightening the most credible locations which are mostly attacked by drugs. The energies of FMOs, for instance HOMO (*E_HOMO_*), LUMO (*E_LUMO_*), as well as energy gaps (Δ*E_HOMO/LUMO_*) between HOMO-LUMO and HOMO-1/LUMO+1 (Δ*E_HOMO/LUMO+1_*), are noteworthy parameters to probe molecules (Table 5).

The chemical potential “*IP*”, nucleophilicity “*χ*”, and electrophilicty “*ω”* were studied using the HOMO–1/LUMO+1 (Table 5).The variations of **Δ*E***_HOMO/LUMO_ for **5**–**18** hybrids is not clear due to the similar energy values. The promising kinase inhibitors were the organic molecule that donates electrons to the positive-charged amino-acids, and able to accept free electrons from kinase (imine groups). The previous studies displayed a direct relation between the potent antioxidant ability and anticancer drugs [33,34]. The antioxidant potential can be assessed by ionization potential (IP). It is expected that the antioxidant ability for **5**–**18** might be better due to low IP values. These results suggest that synthesized **5**–**18** derivatives may have good antioxidant potential.

##### Molecular Electrostatic and Ionization Potential Maps

To explore the reactivity of synthesized hybrids, molecular electrostatic potential (MEP) was measured experimentally by diffraction approaches [35]. This property displays the nuclear and electronic distribution over the synthesized hybrids and is indicated by different colors. The high negative-charge region that attacks by electrophile is indicated by red, whereas the blue region is positively charged, which attacks the nucleophile. MEP decreases in the order green > blue > yellow > orange > red, and the red color shows repulsion while the blue color indicates enough attraction. As anticipated, the green color shields aromatic rings of all molecules, whereas the yellow regions cover the triazole ring. These color changes in the molecules help in the identification of electrophilic and nucleophilic sites for the attack by receptors. The green region denotes high nucleophilicity, making the molecules able to recognize the binding site through ionic interaction between the substrate and receptor. 

**Figure 3:**
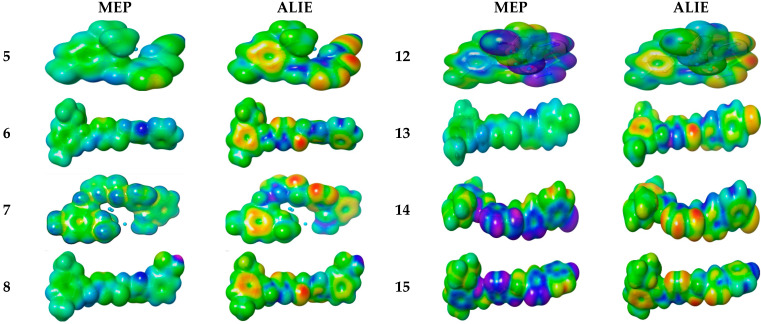

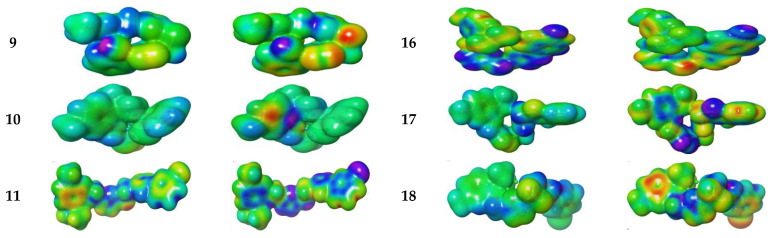
Molecular electrostatic potential surfaces views of **5**–**18.**

The ALIE (average local ionization energy map) was used to predict favorable molecular sites for electrophiles. The red color surrounds the thymol ring in all **6**–**18** (Figure 3). The electron density in the yellow color spreads over all **6**–**18** molecular backbones. The blue color is more localized in the oxadiazole linker. Thereby, these groups are prone to nucleophilic attacks.

#### 2.3.5. Molecular Docking

To know the possible molecular interaction of compounds **5**–**18** *via* thymidylate synthase (TS) and DNA gyrase B, the docking experiment was performed. The 3D structure of TS and DNA gyrase enzymes for anticancer and antimicrobial, respectively, were established. 3D loop structure of PDB 6QXG and 4URO complexed with *FdUMP* and Novobiocin for TS and DNA gyrase, respectively, were used as frameworks for the docking experiment. The 3D models were further checked and verified using Ramachandran plot at PROCHECK [36]. The CASTp Package was used for identifying the active sites for TS and DNA gyrase. TS are reported to bind with Asn 226, His 256, Asp 218, Ser 216, Arg 50, and Arg 215, whereas for DNA gyrase, the binding sites are Arg144, Ser 55, Ala64, Asn65 and Asp89.

##### Thymidylate Synthase (6QXG)

Compounds **5**–**18** were successfully docked over active sites for TS. The generated docked poses were energy-minimized by the OPLS force field, and the lowest-binding free-energy ΔG with minimum RMSD between the pose before and after refinement were selected. Finally, the highest ΔG scoring function was employed to assess the binding energy of the compounds. To further validate the binding energy of **5**–**18**, the inhibition constant (Ki) was calculated. The lower the Ki, better is the efficiency of the molecule, and it should be in the range of 0.1–1.0 μM value. Ligand-Efficiency (LE) and Fit-Quality (FQ) are the bioactivity factors which should be ≥0.3 and ≥0.8, respectively [37]. These parameters were compared with reference inhibitors and are shown in Table 6.

**Figure 4:**
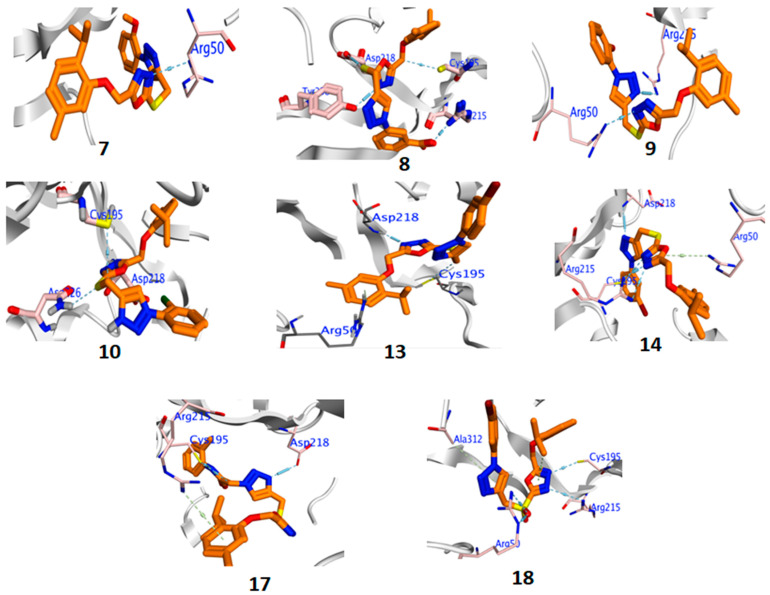
Binding interaction of active compounds against TS (6QXG).

Compound **17** showed a higher glide score than both the reference drugs, fluorodeoxyuridylate (FDUMP) and 5-fluorouracil (FU). Interestingly, all compounds **5**–**18** showed higher BE than FU. RMSDs were found to be <2 degree supporting docking-process accuracy. The BEs for these derivatives were found to be in the order **17** > **FD** > **8** > **14** > **18** > **13** > **6** > **16** > **10** > **11** > **12** > **5** > **FU**. All the compounds **5**–**18** interacted in a similar manner, like FU. These compounds were more potent than the reference drugs, as indicated by lower inhibition constant values (Ki = 0.16–2.16 μM for compounds **5**–**18**; 2.47 and 2.41 μM for reference drugs). All compounds displayed H-bond formation with important amino acid residues of TS (Figure 4). From analysis of the protein-ligand fingerprint PLIF histogram, it was found that residue Arg215 interacted with 50% of those tested by H-bond interactions and Arg50, Glu87, and ASP218 interacted with the remaining 30% of tested compounds (Figure S30, Supplementary Material).

##### DNA Gyrase B (4URO)

Bacterial DNA-gyrase is an attractive antibacterial target, which cleaves the DNA double-helix [38]. The analysis of the co-crystallized DNA-gyrase-novobiocin complex is responsible for restricting the ATPase binding site (Arg144, Ser 55, Ala64, Asn65, Asp89) situated on the cell-wall of organisms. The peptidoglycan layer situated in the cell wall of bacteria inhibits the microorganism *via* antibacterial agents. From Table 7, compounds **5, 6, 7, 9, 16,** and **17** exhibited the highest BE compared with the reference drug, Novobiocin. Other compounds showed lower binding scores with considerable RMSD < 2. These compounds exhibited Ki in the range 1.86–1.99 μM.

**Figure 5:**
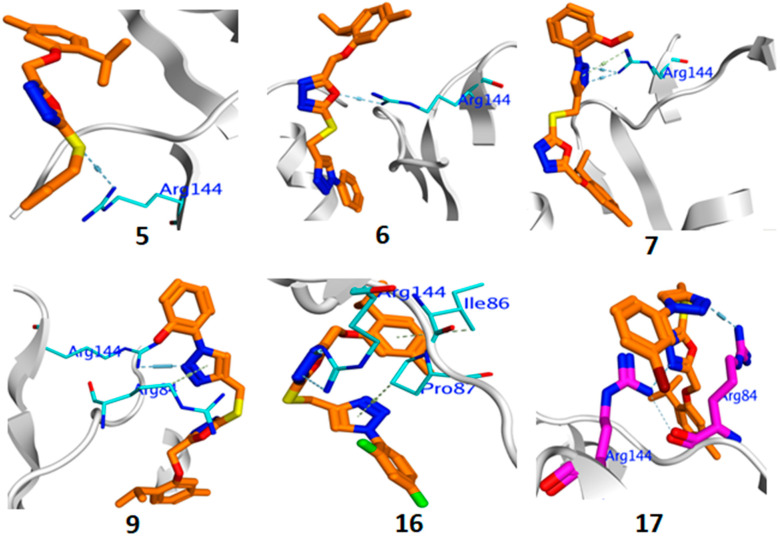
Binding interaction of active compounds against DNA gyrase (4URO).

All the compounds formed H-bonds with vital amino acid residues Glu58, Pro87, Ile86, and Arg144 (Figure 5). PLIF analysis showed the Arg144 interacted through H-bond formation with 43% of the tested compounds (Figure S31, Supplementary Material). The inhibition potency of **6**–**18** against bacterial growth may be explained by the attacking of the peptidoglycan-naked cell-wall of bacteria. Thus, the antimicrobial-mechanism for **5**–**18** hybrids is due to the high permeability of the microorganism membrane. 

## 3. Methods and Materials

### 3.1. Chemistry

The reagents and solvents used for the synthesis were purchased from Sigma Aldrich (St. Louis, MI, USA) and Loba India Pvt. Ltd. (Mumbai, India) and used directly. NMR analysis was performed on Bruker spectrometer (850 MHz) using CDCl_3_ as solvent. IR spectra were recorded on Thermoscientific using ATR technique and melting points were recorded using Stuart SMP 40 machine and were uncorrected. LCQ Fleet-LCF10605 Thermoscientific Mass spectrometer was used to measure the molecular weight of the synthesized compounds and elemental analysis was performed using Elementar Analyzer. Compounds **2, 3,** and **4** were prepared as previously published [30].

Preparation of 2-((2-isopropyl-5-methylphenoxy)methyl)-5-(prop-2-ynylthio)-1,3,4-oxadiazole (**5**)

Compound **4** (0.06 mol) was charged into a 250 mL round-bottom flask, and then anhydrous acetone (175 mL) and potassium carbonate (0.066 mol) were added. The reaction mass was stirred for 2 hrs at 50–60 °C and then cooled to room temperature. To the reaction mixture, propargyl bromide (0.066 mol) was added and the reaction was stirred at 40–50 °C for 4 hrs. After completion of the reaction, the reaction mixture was filtered, and filtrate was concentrated to 30 mL and poured into 100 mL water. The product was isolated by extracting with dichloromethane (2 × 100 mL). To the DCM layer, cyclohexane was added under hot conditions to achieve a solid mass, and the solid product was filtered and washed with cyclohexane (20 mL), and then dried.

Yield: 80%, M.p. 58–60 °C, IR (ATR) υ_max_: 3252 (terminal alkyne C–H), 2985, 2120 (carbob-carbon triple-bond stretching), 1613, 1594, 1507, 1476, 1392, 1383, 1340, 1287 (C=N, C–N), 1176, 1162, 1110, 1096, 1054, 1027 (C–O), 993, 972, 956, 847, 818, 701, 666. ^1^H NMR (CDCl_3_, 850 MHz): δ 1.21 (d, *J* = 7.64 Hz, 6H, –CH(CH_3_)_2_, 2.20 (s, 1H, terminal alkyne H), 2.35 (s, 3H, Aromatic methyl), 3.28-3.31 (m, 1H, –C–H (CH_3_)_2_, 4.04 (s, 2H, S–CH_2_–), 5.26 (s, 2H, –O–CH_2_–), 6.80 (s, 1H), 6.85 (s, 1H), 7.14 (d, J = 8.5 Hz, 1H); ^13^C NMR (CDCl_3_, 213 MHz): δ 21.29, 22.83, 26.49, 30.96, 60.14, 73.09, 112.90, 122.96, 126.45, 134.74, 136.66, 154.63, 164.18, 164.34. ESI MS: 302 (M^+^ + H). C_16_H_18_N_2_O_2_S (Calculated): C = 63.55; H = 6.00; N = 9.26; O = 10.58; S = 10.60; observed: C = 63.52; H = 6.02; N = 9.24; O = 10.62; S = 10.58.

Synthesis of Final Compounds (**6**–**16**)

Compound **5** (0.001 mol) was dissolved in tert-BuOH:water (1:1; 20 mL) in a 250 mL round-bottom flask, and then sodium ascorbate and copper sulphate pentahydrate were added followed by the addition of aromatic azides (0.0011 mole). The reaction mixture was stirred at 20–30 °C for 3–8 h. After completion of the reaction, water (75 mL) and ethylacetate (75 mL) were added to the reaction mixture and stirred for 1 h. The organic layer was separated, washed with water, and dried over anhydrous sodium sulphate. Ethylacetate was evaporated and the compounds were crystallized with a mixture of ethylacetate-cyclohexane or Ethylacetate-hexane.

4-((5-((2-isopropyl-5-methylphenoxy)methyl)-1,3,4-oxadiazol-2-ylthio)methyl)-1-phenyl-1*H*-1,2,3-triazole (**6**)

Yield = 68%, M.p. 88–90 °C, IR (ATR) υ_max_: 3086 (C–H aromatic), 2962 (C–H), 1613, 1593, 1502, 1474, 1413, 1345, 1286, 1254, 1157, 1093, 1053, 1032, 809, 754, 686; ^1^H NMR (850 MHz, CDCl_3_): δ 1.18 (d, *J* = 8.5 Hz, 6H, –CH–(CH_3_)_2_), 2.33 (s, 3H, Aromatic methyl), 3.27-3.29 (m, 1H, –CH–(CH_3_)_2_), 4.69 (s, 2H, S–CH_2_–), 5.22 (s, 2H, –O–CH_2_–), 6.79 (s, 1H), 6.84 (d, *J* = 8.5 Hz, 1H), 7.13 (d, *J* = 8.5 Hz, 1H), 7.46 (t, *J* = 8.5 Hz, 1H), 7.54 (t, *J* = 8.5 Hz, 2H), 7.73 (d, *J* = 8.5 Hz, 2H), 8.46 (s, 1H, triazole ring proton); ^13^C NMR (CDCl_3_, 213 MHz): δ 21.30, 22.84, 26.43, 30.96, 60.19, 112.93, 120.76, 122.99, 126.45, 128.95, 129.85, 134.79, 136.62, 154.61. ESI MS: 422 (M^+^ + H). C_22_H_23_N_5_O_2_S(Calculated): C = 62.69; H = 5.50; N = 16.61; O = 7.59; S = 7.61; observed: C = 62.52; H = 5.54; N = 16.64; O = 7.62; S = 7.58.

4-((5-((2-isopropyl-5-methylphenoxy)methyl)-1,3,4-oxadiazol-2-ylthio)methyl)-1-(2-methoxyphenyl)-1*H*-1,2,3-triazole (**7**) 

Yield = 65%, M.p. 81-83 °C, IR (ATR) υ_max_: 2960, 1614, 1586, 1558, 1540, 1505, 1473, 1417, 1391, 1360, 1289, 1255, 1242 (C=N, C–N), 1159, 1095, 1045, 1027, 813, 786, 751, 741, 724, 674; ^1^H NMR (850 MHz, CDCl_3_): δ 1.19 (d, *J* = 8.5 Hz, 6H, –CH–(CH_3_)_2_), 2.33 (s, 3H, Aromatic methyl), 3.27-3.30 (m, 1H, –CH–(CH_3_)_2_), 3.88 (s, 3H, O–CH_3_), 4.73 (s, 2H, S–CH_2_–), 5.23 (s, 2H, –O–CH_2_–), 6.80 (s, 1H), 6.84 (d, *J* = 8.5 Hz, 1H), 7.10-7.14 (m, 3H), 7.44 (t, *J* = 8.5 Hz, 1H), 7.81 (s, 1H), 8.50 (s, 1H, triazole ring proton); ^13^C NMR (CDCl_3_, 213 MHz): δ 21.31, 22.84, 26.43, 30.96, 55.94, 60.20, 112.29, 112.93, 121.24, 122.96, 126.44, 130.28, 134.79, 136.61, 154.64. ESI MS: 452 (M^+^ + H). C_23_H_25_N_5_O_2_S (Calculated): C = 61.18; H = 5.58; N = 15.51; O = 10.63; S = 7.10; observed: C = 61.14; H = 5.60; N = 15.49; O = 10.65; S = 7.09.

3-(4-((5-((2-isopropyl-5-methylphenoxy)methyl)-1,3,4-oxadiazol-2-ylthio)methyl)- 1*H*-1,2,3-triazol-1-yl)benzoic acid (**8**) 

Yield = 73%, M.p. 166-168 °C, IR (ATR) υ_max_: 3120 (COO–H), 2995 (C–H), 1700 (C=O), 1614, 1591, 1493, 1473, 1411, 1252, 1161, 1116, 1095, 1051, 1000, 952, 902, 858, 811, 792, 768, 691. ^1^H NMR (850 MHz, CDCl_3_): δ 1.18 (d, *J* = 8.5 Hz, 6H, –CH–(CH_3_)_2_), 2.33 (s, 3H, Aromatic methyl), 3.27–3.30 (m, 1H, –CH–(CH_3_)_2_), 4.74 (s, 2H, S–CH_2_–), 5.21 (s, 2H, –O–CH_2_–), 6.80 (s, 1H), 6.84 (d, *J* = 8.5 Hz, 1H), 7.13 (d, J = 8.5 Hz, 1H), 7.68 (t, J = 8.5 Hz, 1H), 8.10 (s, 1H), 8.21 (d, *J* = 8.5 Hz, 1H), 8.48 (s, 1H, triazole ring proton); ^13^C NMR (CDCl_3_, 213 MHz): δ 21.32, 22.84, 26.43, 30.96, 60.17, 112.93, 123.01, 126.45, 130.38, 134.79, 136.64, 154.58, 168.76. ESI MS: 464 (M^+^ + H). C_23_H_23_N_5_O_4_S (Calculated): C = 59.34; H = 4.98; N = 15.04; O = 13.75; S = 6.89; observed: C = 59.24; H = 4.99; N = 15.02; O = 13.78; S = 6.86.

2-(4-((5-((2-isopropyl-5-methylphenoxy)methyl)-1,3,4-oxadiazol-2-ylthio)methyl)- 1*H*-1,2,3-triazol-1-yl)phenol (**9**) 

Yield = 70%, M.p. 86–88 °C, IR (ATR) υ_max_: 3000 (brd peak, O–H), 2952 (C–H), 1599, 1506, 1472, 1424, 1405, 1290, 1261, 1167, 117, 1096, 1063, 1042 (C–O), 989, 943, 806, 792, 752, 718, 642; ^1^H NMR (850 MHz, CDCl_3_): δ 1.19 (d, *J* = 8.5 Hz, 6H, –CH–(CH_3_)_2_), 2.34 (s, 3H, Aromatic methyl), 3.27-3.30 (m, 1H, –CH–(CH_3_)_2_), 4.71 (s, 2H, S–CH_2_–), 5.22 (s, 2H, –O–CH_2_–), 6.80 (s, 1H), 6.84 (d, *J* = 8.5 Hz, 1H), 7.13 (d, *J* = 8.5 Hz, 1H), 7.24 (d, *J* = 8.5 Hz, 1H), 7.33 (d, *J* = 7.5 Hz, 1H), 7.42 (d, *J* = 8.5 Hz, 1H), 9.67 (s, 1H, phenolic proton); ^13^C NMR (CDCl_3_, 213 MHz): δ 21.32, 22.84, 26.44, 30.96, 60.19, 112.93, 119.55, 120.52, 123.02, 126.47, 129.93, 143.79, 136.64, 149.18, 154.59. ESI MS: 438 (M^+^ + H). C_22_H_23_N_5_O_3_S (Calculated): C = 60.39; H = 5.30; N = 16.01; O = 10.97; S = 7.33; observed: C = 60.35; H = 5.32; N = 15.98; O = 10.99; S = 7.32.

4-((5-((2-isopropyl-5-methylphenoxy)methyl)-1,3,4-oxadiazol-2-ylthio)methyl)-1-(2-chlorophenyl)-1*H*-1,2,3-triazole (**10**) 

Yield = 75%, M.p. 90–92 °C, IR (ATR) υ_max_: 2960, 1594, 1505, 1485, 1437, 1287, 1253, 1157, 1116, 1095, 1042, 812, 784, 679.^1^H NMR (850 MHz, CDCl_3_): δ 1.17–2.20 (m, 6H, –CH–(CH_3_)_2_), 2.34 (s, 3H, Aromatic methyl), 3.29 (brd, s, 1H, –CH–(CH_3_)_2_), 4.64 (s, 2H, S–CH_2_–), 5.25 (s, 2H, –O–CH_2_–), 6.81–6.85 (m, 2H), 7.14 (d, *J* = 8.5 Hz, 1H), 7.46–7.82 (m, 4H); ^13^C NMR (CDCl_3_, 213 MHz): δ 21.32, 22.85, 26.44, 30.96, 60.23, 112.95, 123.02, 122.99, 126.47, 128.97, 134.79, 143.80, 136.63, 155.50. ESI MS: 456 (M^+^ + H), 458 (M^+^ + H+2). C_22_H_22_ClN_5_O_2_S (Calculated): C = 57.95; H = 4.86; N = 15.36; O = 7.02; S = 7.03; observed: C = 57.90; H = 4.88; N = 15.34; O = 7.04; S = 7.01.

4-((5-((2-isopropyl-5-methylphenoxy)methyl)-1,3,4-oxadiazol-2-ylthio)methyl)-1-(3-chlorophenyl)-1*H*-1,2,3-triazole (**11**) 

Yield = 68%, M.p. 92–94 °C, IR (ATR) υ_max_: 2962 (C–H), 1614, 1592, 1495, 1475, 1416, 1380, 1344, 1285, 1251, 1158, 1112, 1098, 1074, 807, 752; ^1^H NMR (850 MHz, CDCl_3_): δ 1.18 (d, *J* = 8.5 Hz, 6H, –CH–(CH_3_)_2_), 2.33 (s, 3H, Aromatic methyl), 3.26-3.29 (m, 1H, –CH–(CH_3_)_2_), 4.69 (s, 2H, S–CH_2_–), 5.23 (s, 2H, –O–CH_2_–), 6.78 (s, 1H), 6.83 (d, *J* = 8.5 Hz, 1H), 7.13 (d, *J* = 8.5 Hz, 1H), 7.45–7.48 (m, 2H), 7.59–7.62 (m, 2H), 8.18 (s, 1H); ^13^C NMR (CDCl_3_, 213 MHz): δ 21.29, 22.83, 26.44, 26.82, 30.96, 60.15, 112.90, 122.96, 125.51, 126.44, 127.76, 127.94, 128.85, 130.82, 130.90, 134.71, 134.76, 136.62, 146.12,154.60. ESI MS: 456 (M^+^ + H), 458 (M^+^ + H+2).C_22_H_22_ClN_5_O_2_S (Calculated): C = 57.95; H = 4.86; N = 15.36; O = 7.02; S = 7.03; observed: C = 57.91; H = 4.88; N = 15.33; O = 7.04; S = 7.02.

4-((5-((2-isopropyl-5-methylphenoxy)methyl)-1,3,4-oxadiazol-2-ylthio)methyl)-1-(2,4-dichlorophenyl)-1*H*-1,2,3-triazole (**12**) 

Yield = 78%, M.p. 84–86 °C, IR (ATR) υ_max_: 2961 (C–H), 1614, 1594, 1505, 1494, 1251, 1169, 1154, 1099, 999, 813, 778, 743, 730, 682. ^1^H NMR (850 MHz, CDCl_3_): δ 1.19 (d, *J* = 8.5 Hz, 6H, –CH–(CH_3_)_2_), 2.33 (s, 3H, Ar-CH_3_), 3.26–3.30 (m, 1H, –CH–(CH_3_)_2_), 4.71 (s, 2H, S–CH_2_–), 5.22 (s, 2H, –O–CH_2_–), 6.79–6.83 (m, 2H), 7.13 (d, *J* = 8.5 Hz, 1H), 7.44(d, *J* = 8.5 Hz, 1H), 7.57–7.62 (m, 2H), 8.45 (s, 1H); ^13^C NMR (CDCl_3_, 213 MHz): δ 21.33, 22.84, 26.45, 30.96, 60.16, 112.90, 122.99, 126.45, 128.33, 128.44, 129.51, 130.68, 134.75, 136.49, 136.63, 154.58, ESI MS: 490 (M^+^ + H), 492 (M^+^ + H+2).C_22_H_21_Cl_2_N_5_O_2_S (Calculated): C = 53.88; H = 4.32; N = 14.28; O = 6.52; S = 6.54; observed: C = 53.82; H = 4.34; N = 14.26; O = 6.53; S = 6.51.

4-((5-((2-isopropyl-5-methylphenoxy)methyl)-1,3,4-oxadiazol-2-ylthio)methyl)-1-(4-bromophenyl)-1*H*-1,2,3-triazole (**13**) 

Yield = 80%, M.p. 114–116 °C, IR (ATR) υ_max_: 2963 (C–H), 1615, 1592, 1497, 1476, 1414, 1379, 1285, 1254, 1190, 1153, 1091, 1075, 1045, 986, 817, 809; ^1^H NMR (850 MHz, CDCl_3_): δ 1.19 (d, *J* = 8.5 Hz, 6H, –CH–(CH_3_)_2_), 2.34 (s, 3H, Ar-CH_3_), 3.27–3.29 (m, 1H, –CH–-(CH_3_)_2_), 4.72 (s, 2H, S–CH_2_–), 5.20 (s, 2H, –O–CH_2_–), 6.77–6.85 (m, 2H), 7.13 (d, *J* = 8.5 Hz, 1H), 7.66 (s, 3H), 8.5 (s, 1H); ^13^C NMR (CDCl_3_, 213 MHz): δ 21.35, 22.85, 26.45, 30.96, 60.22, 112.93, 122.65, 123.03, 126.47, 128.97, 133.18, 134.78, 136.65, 154.59.ESI MS: 500 (M^+^ + H), 502 (M^+^ + H+2).C_22_H_22_BrN_5_O_2_S (Calculated): C = 52.80; H = 4.43; N = 14.00; O = 6.39; S = 6.41; observed: C = 52.74; H = 4.45; N = 14.02; O = 6.41; S = 6.39.

4-((5-((2-isopropyl-5-methylphenoxy)methyl)-1,3,4-oxadiazol-2-ylthio)methyl)-1-(3-bromophenyl)-1*H*-1,2,3-triazole (**14**)

Yield = 75%, M.p. 72–74 °C, IR (ATR) υ_max_: 3091C-H, aromatic), 2958 (C–H), 1614, 1588, 1506, 1493, 1473, 1435, 1252, 1160, 1095, 1051, 1041, 952, 942, 859, 812, 792, 768, 673; ^1^H NMR (850 MHz, CDCl_3_): δ 1.19 (d, *J* = 8.5 Hz, 6H, –CH–(CH_3_)_2_), 2.34 (s, 3H, Aromatic methyl), 3.27–3.30 (m, 1H, –CH–(CH_3_)_2_), 4.70 (s, 2H, S–CH_2_–), 5.20 (s, 2H, –O–CH_2_–), 6.80 (s, 1H), 6.84 (d, *J* = 8.5 Hz, 1H), 7.13 (d, *J* = 8.5 Hz, 1H), 7.41–7.42 (m, 2H), 7.60 (d, *J* = 8.5 Hz, 1H), 7.70 (d, *J* = 8.5 Hz, 1H), 7.97 (s, 1H), 8.58 (s, 1H); ^13^C NMR (CDCl_3_, 213 MHz): δ 21.34, 22.85, 26.44, 30.96, 60.19, 112.93, 123.02, 123.60, 124.01, 126.47, 131.28, 131.96, 134.79, 136.64, 154.60. ESI MS: 500 (M^+^ + H), 502 (M^+^ + H+2). C_22_H_22_BrN_5_O_2_S (Calculated): C = 52.80; H = 4.43; N = 14.00; O = 6.39; S = 6.41; observed: C = 52.77; H = 4.45; N = 14.02; O = 6.41; S = 6.39.

4-((5-((2-isopropyl-5-methylphenoxy)methyl)-1,3,4-oxadiazol-2-ylthio)methyl)-1-(4-florophenyl)-1*H*-1,2,3-triazole (**15**) 

Yield = 75%, M.p. 118–120 °C, IR (ATR) υ_max_: 3090, 2958, 2868, 1614, 1588, 1506, 1492, 1473, 1348, 1288, 1252, 1160, 1095, 1051, 1000, 952, 942, 859, 812, 792, 768, 749, 673, 591,^1^H NMR (850 MHz, CDCl_3_): δ 1.19 (d, *J* = 8.5 Hz, 6H, –CH–(CH_3_)_2_), 2.33 (s, 3H, Aromatic methyl), 3.27–3.29 (m, 1H, –CH–(CH_3_)_2_), 4.67 (s, 2H, S–CH_2_–), 5.23 (s, 2H, –O–CH_2_–), 6.80–6.84 (m, 2H), 7.13 (d, *J* = 8.5 Hz, 1H), 7.22–7.24 (m, 2H), 7.71 (brd, s, 2H), 8.31 (s, 1H); ^13^C NMR (CDCl_3_, 213 MHz): δ 21.30, 22.84, 26.44, 30.96, 60.19, 112.93, 116.80, 122.80, 123.01, 126.46, 134.78, 136.64, 154.59, 161.95, 163.12.ESI MS: 440 (M^+^ + H).C_22_H_22_FN_5_O_2_S (Calculated): C = 60.12; H = 5.05; N = 15.93; O = 7.28; S = 7.30; observed: C = 60.08; H = 5.07; N = 15.90; O = 7.30; S = 7.31.

4-((5-((2-isopropyl-5-methylphenoxy)methyl)-1,3,4-oxadiazol-2-ylthio)methyl)-1-(2,4-diflorophenyl)-1*H*-1,2,3-triazole (**16**)

Yield = 75%, M.p. 130–132 °C, IR (ATR) υ_max_: 3096 (C–H, aromatic), 2965 (C–H), 1615, 1519, 1506, 1473, 1455, 1284, 1254,1157, 1109, 1093, 1055, 1045, 958, 849, 810, 689, 609. ^1^H NMR (850 MHz, CDCl_3_): δ 1.19 (d, *J* = 8.5 Hz, 6H, –CH–-(CH_3_)_2_), 2.33 (s, 3H, Aromatic methyl), 3.29 (brd, s, 1H, –CH–(CH_3_)_2_), 4.72 (s, 2H, S–CH_2_–), 5.22 (s, 2H, –O–CH_2_–), 6.80–6.84 (m, 2H), 7.08–7.14 (m, 3H), 7.93 (s, 1H), 8.60 (s, 1H); ^13^C NMR (CDCl_3_, 213 MHz): δ 21.34, 22.84, 26.45, 30.97, 60.21, 105.55, 112.92, 122.99, 126.45, 134.77, 136.64, 154.61, 161.98, 163.21. ESI MS: 458 (M^+^ + H). C_22_H_21_F_2_N_5_O_2_S (Calculated): C = 57.76; H = 4.63; N = 15.31; O = 6.99; S = 7.01; observed: C = 57.71; H = 4.65; N = 15.28; O = 7.03; S = 7.00.

Preparation of 4-((5-((2-isopropyl-5-methylphenoxy)methyl)-1,3,4-oxadiazol-2-ylthio)methyl)-N-*o*-tolyl-1*H*-1,2,3-triazole-1-carboxamide (**17**)

Compound **17** was prepared according to same methods as used for compounds **6–16**. Yield = 60%, M.p. 124–126 °C, IR (ATR) υ_max_: 3131 (N–H), 3091 (C–H, aromatic), 2958, 1653, 1614, 1588, 1507, 1493, 1435, 1411, 1349, 1288, 1252, 1160, 1095, 1051, 1039, 993, 963, 859, 812, 792, 767, 673. ^1^H NMR (850 MHz, CDCl_3_): δ 1.19 (d, *J* = 8.5 Hz, 6H, –CH–(CH_3_)_2_), 2.34 (m, 6H, Aromatic methyl), 3.29 (brd, s, 1H, –CH–(CH_3_)_2_), 4.72 (s, 2H, S–CH_2_–), 5.24 (s, 2H, –O-CH_2_–), 6.80-6.82 (m, 2H), 7.10–7.14 (m, 4H), 7.93 (s, 1H), 8.60 (s, 1H); ESI MS: 479 (M^+^ + H). C_25_H_28_N_6_O_3_S (Calculated): C = 60.96; H = 5.73; N = 17.06; O = 9.74; S = 6.51; observed: C = 60.92; H = 5.76; N = 17.04; O = 9.76; S = 6.49.

Preparation of 4-((5-((2-isopropyl-5-methylphenoxy)methyl)-1,3,4-oxadiazol-2-ylsulfonyl)methyl)-1-(3-bromophenyl)-1*H*-1,2,3-triazole (**18**)

Compound **14** (0.0005 mole) was added into a 100 mL round-bottom flask followed by the addition of dichloromethane (100 mL) and hydrogen peroxide (20 mL, 30%). The reaction mass was stirred for 28 hrs. After completion of the reaction, the reaction mixture was added into 100 mL water and extracted with DCM (50 mL). The DCM layer was concentrated and product was crystallized with cyclohexane and DCM.

Yield = 64%, M.p.98–100 °C, IR (ATR) υ_max_: 2962, 1614, 1591, 1533, 1498, 1476, 1456, 1413, 1379, 1287, 1253, 1155, 1113, 1093, 1046, 986, 810, 779, 752, 688, 593. ^1^H NMR (850 MHz, CDCl_3_): δ 1.19 (d, *J* = 8.5 Hz, 6H, –CH–(CH_3_)_2_), 2.34 (s, 3H, Aromatic methyl), 3.26-3.30 (m, 1H, –CH–(CH_3_)_2_), 4.96 (s, 2H, S–CH_2_–), 5.20 (s, 2H, –O–CH_2_–), 6.80 (s, 1H), 6.82 (d, *J* = 8.5 Hz, 1H), 7.12 (d, *J* = 8.5 Hz, 1H), 7.41-7.42 (m, 2H), 7.60 (d, *J* = 8.5 Hz, 1H), 7.68 (d, *J* = 8.5 Hz, 1H), 7.97 (s, 1H), 8.58 (s, 1H); ESI MS: 533 (M^+^ + H), 535 (M^+^ + H+2). C_22_H_22_BrN_5_O_4_S (Calculated): C = 49.63; H = 4.16; N = 13.15; O = 12.02; S = 6.02; observed: C = 49.60; H = 4.17; N = 13.13; O = 12.05; S = 6.01.

### 3.2. Biological Activities

#### 3.2.1. Antiproliferative Activity 

The antiproliferative activity of the final compounds **6**–**18** were performed by MTT method as previously published [26]. The compounds were screened on breast (MCF-7), liver (HepG2), and colorectal (HCT-116) cancer cells, obtained from American Type culture Collection (ATCC); 0.1% of DMSO was used as a vehicle control [26]. 

#### 3.2.2. Thymidylate Synthase Activity

The activity was carried out as previously published [26].

#### 3.2.3. Antimicrobial Activity

The antimicrobial activity of the final compounds was performed by reported methods [39].The bacterial strains, *E. coli* (Gram negative), *S. aureus* (Gram positive), and fungi, *Candida albicans*, were used for the study. Luria broth Agar and Sabouraud Dextrose Agar were prepared into a petri dish using 25 mL of autoclaved media; 100 μL of diluted broth of freshly prepared *E. coli*, *S. aureus,* and *C. albicans* were spread on Luria Broth Agar and Sabouraud Agar plates, respectively. Then, **7** wells of 50 μL capacity were punched on each plate. Samples **5**–**18** (10 μL of 10 mg/mL standard prepared in 50% DMSO) were placed in each plate’s well in different plate sets. All the plates were incubated for overnight at 37 °C in an incubator shaker. The inhibition zone was calculated in triplicate. The results are shown in Table 4.

#### 3.2.4. Statistical Analysis

Data are presented as mean standard deviation unless otherwise indicated. Significance of the statistical analysis was acceptable to a level of *p* < 0.05. 

### 3.3. Computational Details and Molecular Docking

The computational studies were carried out by Jaguar package for DFT calculations. The docking studies were performed on thymidylate synthase (PDB 6QXG) and DNA gyrase (PDB 4URO) for anticancer and antimicrobial studies, respectively. The simulation and docking studies were performed according to our previous published work [20].

## 4. Conclusions

A library of synthesized 1,2,3-triazole-incorporated thymol-1,3,4-oxadiazole derivatives (**6**–**18**) was tested for anticancer and antimicrobial activities. Compounds **7**, **8**, **9**, **10,** and **11** exhibited significant antiproliferative activity. Among these active derivatives, compound 2-(4-((5-((2-isopropyl-5-methylphenoxy)methyl)-1,3,4-oxadiazol-2-ylthio)methyl)-1*H*-1,2,3-triazol-1-yl)phenol (**9**)was the best compound against all three tested cell lines, MCF-7 (IC_50_ 1.1 μM), HCT-116 (IC_50_ 2.6 μM), and HepG2 (IC_50_ 1.4 μM). Compound **9** was found to be better than the standard drugs, doxorubicin and 5-fluorouracil. These compounds showed anticancer activity through thymidylate synthase inhibition, as they displayed significant TS inhibitory activity with IC_50_ in the range 1.95–4.24 μM, whereas the standard drug, Pemetrexed, showed IC_50_ 7.26 μM. The antimicrobial results showed that some of the compounds (**6, 7, 9, 16,** and **17**) exhibited good inhibition on *E. coli* and *S. aureus*. The molecular docking and simulation studies supported the anticancer and antimicrobial data. It can be concluded that the synthesized 1,2,3-triazole tethered thymol-1,3,4-oxadiazole conjugates have both antiproliferative and antimicrobial potential.

## Figures and Tables

**Table 1:** Absorption, distribution, metabolism, and elimination (ADME)/Pharmacokinetics studies of final hybrids **5**–**18.**

No.	Lipinski Parameters					nROTB ^e^	TPSA ^f^	%ABS ^g^	BBB ^h^	GI ABS ^i^
	MW ^a^	HBA ^b^	HBD ^c^	iLogP ^d^	Violations					
5	302.39	4	0	3.61	0	6	73.45	83.65	Yes	High
6	421.52	6	0	4.14	0	8	104.16	73.07	No	High
7	451.54	7	0	4.54	1	9	113.39	69.88	No	High
8	465.52	8	1	3.93	0	9	141.46	60.20	No	Low
9	437.51	7	1	4.08	0	8	124.39	66.09	No	Low
10	455.96	6	0	4.22	0	8	104.16	73.07	No	High
11	455.96	6	0	4.05	0	8	104.16	73.07	No	High
12	490.41	6	0	4.45	1	8	104.16	73.07	No	Low
13	500.41	6	0	4.61	1	8	104.16	73.07	No	High
14	500.41	6	0	4.19	1	8	107.16	73.07	No	High
15	439.51	7	0	4.29	0	8	104.16	73.07	No	High
16	457.50	8	0	4.30	1	8	104.16	73.07	No	Low
17	492.59	7	1	3.26	0	11	133.26	63.02	No	Low
18	532.41	8	0	3.99	1	8	121.38	67.13	No	Low

^a^: Molecular weight; ^b^: Hydrogen bond acceptor; ^c^: Hydrogen bond donor; ^d^: Lipophilicity; ^e^: Number of rotatable bonds; ^f^: Topological polar surface area; ^g^: Absorption percentage; calculated by %ABS = 109 − (0.345 × TPSA); ^h^: blood-brain barrier permeability; ^i^: Gastrointestinal absorption.

**Table 2:** Cytotoxicity (IC_50_ ± SD, μM) of the final derivatives against MCF-7, HCT-116, and HepG2 cell lines.

Compound	MCF-7	HCT-116	HepG2
**6**	22.5	36.5	41.6
**7**	2.4	3.1	1.8
**8**	3.9	5.8	5.3
**9**	1.1	2.6	1.4
**10**	1.3	3.8	2.5
**11**	4.9	16.3	18.6
**12**	59.4	47.3	40.6
**13**	65.3	78.7	69.4
**14**	41.8	32.8	23.7
**15**	23.15	31.4	49.2
**16**	34.5	56.2	58.3
**17**	9.39	17.3	39.1
**18**	98.2	118.5	95.7
Dox	1.2	2.5	1.8
5-FU	18.74	30.68	28.65

Values are the mean ± SD of three different experiments. Dox-doxorubicin and 5-FU-5-florouracil (reference drug).

**Table 3:** Thymidylate synthase (TS) activity of the potent derivatives.

Compounds	IC_50_ (µM)
**7**	3.52 ± 1.2
**8**	3.98 ± 1.1
**9**	1.95 ± 0.9
**10**	2.18 ± 1.7
**11**	4.24 ± 0.8
Pemetrexed	7.26 ± 1.1

IC_50_ values are the mean ± S.D. of three separate experiments. Pemetrexed (positive control).

**Table 4:** Antimicrobial activity of compounds **5**–**18.**

Compound No.	*E. coli*	*S. aureus*	*C. albicans*
**5**	12 ± 0.32	9 ± 0.30	10 ± 0.32
**6**	14 ± 0.38	10 ± 0.40	---
**7**	12 ± 0.34	12 ± 0.34	---
**8**	---	10 ± 0.40	---
**9**	12 ± 0.36	12 ± 0.34	10 ± 0.032
**10**	---	---	---
**11**	---	---	---
**12**	10 ± 0.38	---	12 ± 0.036
**13**	11 ± 0.34	---	---
**14**	9 ± 0.32	---	---
**15**	---	---	---
**16**	14 ± 0.34	15±0.38	---
**17**	12 ± 0.32	12 ± 0.34	---
**18**	---	---	---
Novobiocin	14 ± 0.64	10 ± 0.24	NT
Fluconazole	NT	NT	16 ± 0.60

--- = no zone of inhibition; NT = not tested; *E. coli* (ATCC 31218); *S. aureus* (ATCC 29213); *C. albicans* (ATCC 10231).

**Table 5:** The calculated energy for **5**–**18** derivatives.

Compd.	E _HOMO_	E _LUMO_	ΔE _HOMO/LUMO_	E _HOMO-1_	E _LUMO+1_	ΔE _HOMO-1/LUMO+1_	IP	η	S	χ	ω
**5**	−6.026	−1.802	−4.224	−0.150	−0.062	−0.0881	5.83	2.612	0.383	2.231	−3.414
**6**	−5.834	−1.472	−4.362	−1.469	−0.707	−0.7619	5.72	2.181	0.458	3.059	−3.653
**7**	−5.995	−1.200	−4.795	−1.360	−1.159	−0.215	1.87	2.398	0.417	2.699	−3.597
**8**	−5.836	−1.987	−3.849	−1.361	−0.424	−0.9361	5.71	1.924	0.520	3.976	−3.912
**9**	−6.104	−1.227	−4.877	−0.963	−0.435	−0.5279	5.82	2.439	0.410	2.755	−3.666
**10**	−6.139	−1.368	−4.771	−0.931	−0.844	−0.0871	6.11	2.386	0.419	2.952	−3.753
**11**	−5.904	−1.794	−4.110	−1.083	−0.615	−0.4680	5.69	2.055	0.487	3.604	−3.849
**12**	−6.181	−1.588	−4.593	−1.197	−0.990	−0.2068	5.67	2.296	0.435	3.285	−3.884
**13**	−5.861	−1.739	−4.122	−1.069	−0.459	−0.6106	5.67	2.061	0.485	3.502	−3.800
**14**	−5.892	−1.828	−4.064	−1.140	−0.552	−0.5875	5.90	2.032	0.492	3.666	−3.860
**15**	−5.849	−1.516	−4.332	−1.026	−0.422	−0.6041	5.77	2.166	0.462	3.130	−3.683
**16**	−6.218	−1.473	−4.745	−1.031	−1.004	−0.0272	6.07	2.372	0.422	3.117	−3.846
**17**	−5.961	−1.277	−4.684	−0.917	−0.705	−0.2122	5.92	2.342	0.427	2.796	−3.619
**18**	−5.989	−1.821	−4.168	−1.625	−1.059	−0.5660	5.70	2.084	0.480	3.659	−3.905

**Table 6:** Docking energy in (kcal/mol) and bioactivity analysis parameters against 6QXG.

Cpd.	ΔE	RMSD	Eplace	Econf	Eele	LE	Ki	LE_scale_	FQ
FdUMP	−7.90	0.94	−430.17	−15.64	−14.74	−8.39	2.47	3.30	5.09
5FU	−5.28	1.60	−433.91	−21.32	−17.47	−3.30	2.41	−5.28	−5.28
**5**	−6.42	1.26	2.48	−14.45	−9.31	−5.08	1.98	3.05	2.03
**6**	−7.36	1.64	41.66	−17.27	−10.34	−4.48	1.54	2.82	1.67
**7**	−7.50	1.10	39.34	−17.66	−8.68	−6.81	2.16	3.17	3.64
**8**	−7.59	1.92	19.80	−7.35	−7.87	−3.95	1.73	2.68	1.27
**9**	−7.46	1.63	32.10	−8.59	−9.55	−4.56	1.94	2.82	1.74
**10**	−7.14	1.01	20.09	−14.91	−7.02	−7.04	1.56	3.24	3.80
**11**	−7.04	1.53	36.13	−14.00	−7.68	−4.61	0.16	2.88	1.73
**12**	−6.87	1.73	35.84	−18.02	−9.06	−3.97	1.81	2.77	1.20
**13**	−7.39	1.40	48.40	−17.34	−12.24	−5.27	1.43	2.96	2.32
**14**	−7.50	1.33	40.76	−12.81	−8.17	−5.63	1.49	3.00	2.63
**15**	−7.04	1.46	43.98	−23.03	−10.03	−4.82	0.94	2.92	1.90
**16**	−7.17	1.65	32.20	−10.86	−9.15	−4.36	1.88	2.81	1.54
**17**	−8.16	1.60	−4.51	−17.16	−8.75	−5.10	2.03	2.84	2.26
**18**	−7.45	1.56	131.24	−15.92	−10.20	−4.78	1.77	2.86	1.91

ΔE, final free-binding energy; Econf, binding energy for the ligand-conformer; Eplace, binding energy of the ligand-receptor; E.ele, ligand-receptor Electrostatic interaction; RMSD, the root mean square deviation of the pose of the docking pose compared to the co-crystal ligand position, LE; Ligand efficiency; Ki-consequent inhibition constant (μM).

**Table 7:** Docking energy in (kcal/mol) and bioactivity analysis parameters against 4URO.

Cpd.	ΔE	RMSD	Eplace	Econf	Eele	LE	Ki	LE_scale_	FQ
Nov.	−8.41	1.16	10.98	−9.99	−7.80	−7.24	1.95	3.56	8.26
**5**	−9.18	1.97	48.83	−10.04	−8.81	−4.67	1.86	3.12	4.12
**6**	−9.57	1.11	57.74	−5.82	−2.67	−8.65	1.82	2.66	2.01
**7**	−8.95	1.58	28.83	−12.31	−12.87	−5.66	1.89	3.16	5.49
**8**	−8.26	1.19	32.72	−5.94	−6.46	−6.93	1.97	2.85	2.81
**9**	−9.26	1.94	31.38	−9.99	−9.03	−4.77	1.86	3.10	3.83
**10**	−6.09	1.79	31.82	−43.28	−2.51	−1.17	3.34	2.67	2.10
**11**	−8.49	1.38	60.48	−6.87	−1.93	−6.14	1.94	2.74	1.57
**12**	−8.21	1.96	48.99	−6.93	−6.42	−4.19	1.98	2.97	3.17
**13**	−8.26	1.87	41.56	−9.63	−7.28	−4.42	1.97	2.66	1.52
**14**	−6.09	0.82	41.09	−41.70	−2.56	−2.56	1.96	2.70	1.72
**15**	−8.08	1.23	30.40	−8.24	−6.28	−6.58	1.99	3.43	0.86
**16**	−9.62	1.26	14.00	−5.65	−9.83	−7.61	1.82	3.07	3.51
**17**	−8.68	1.48	129.68	−7.59	−10.45	−5.85	1.92	3.05	4.56
**18**	−8.26	0.70	44.95	−22.82	−12.90	−11.83	1.97	2.91	2.95

ΔE, final free-binding energy; Econf, binding energy for the ligand-conformer; Eplace, binding energy of the ligand-receptor; E.ele, ligand-receptor Electrostatic interaction; RMSD, the root mean square deviation of the pose of the docking pose compared to the co-crystal ligand position, LE; Ligand efficiency; Ki-consequent inhibition constant (μM), Nov., Novobiocin (positive control).

## Data Availability

Data is contained within the article and supplementary material.

## Supplementary Materials

The following are available online at https://www.mdpi.com/article/10.3390/ph14090866/s1, Figures S1–S9: Proton (1H) NMR of final derivatives; Figures S10–S18: Carbon (13C) NMR of final derivatives; Figures S19–S29: Mass spectra of final derivatives; Figure S30: PLIF histogram with thymidylate synthase; Figure S31: PLIF histogram with DNA gyrase B.8.5H.
