# Supplementary material for: Synthesis and Biological Evaluation of 1,2,3-Triazole Tethered Thymol-1,3,4-Oxadiazole Derivatives as Anticancer and Antimicrobial Agents
Source: Pharmaceuticals (Basel). 2021 Aug 28;14(9):866. doi: 10.3390/ph14090866 (PMC8468421; doi:10.3390/ph14090866)
Supplement: Supplementary file 1 [file pharmaceuticals-14-00866-s001.zip › pharmaceuticals-1353274-supplementary.pdf]

SUPPLEMENTARY MATERIAL

Synthesis and Biological evaluation of 1,2,3-triazole tethered thymol-1,3,4-oxadiazole derivatives as anticancer and antimicrobial agents

Figure S1-S9: ^{1}H NMR of final compounds

Compound 6

4/23/2021 9:41:27 PM

Acquisition Time (sec)	1.9268	*Comment*	Dr. Abdusattar Sampe 11-04 CDCL3			*Date*	20 Apr 2021 16:02:56
Date Stamp	20 Apr 2021 16:02:56						
File Name	D:\NMR Thymol oxadiazole triazole\Azizah_20210422\ABDUSATTAR_11-04_M2_20-04-2021\20\fid					*Frequency (MHz)*	850.15
Nucleus	1H	*Number of Transients*	32	*Origin*	spect	*Original Points Count*	32768
Owner	nmr	*Points Count*	32768	*Pulse Sequence*	zg30	*Receiver Gain*	11.37
SW(cyclical) (Hz)	17006.80	*Solvent*	CHLOROFORM-d			*Spectrum Offset (Hz)*	5250.0283
Spectrum Type	STANDARD	*Sweep Width (Hz)*	17006.28	*Temperature (degree C)*	24.999		

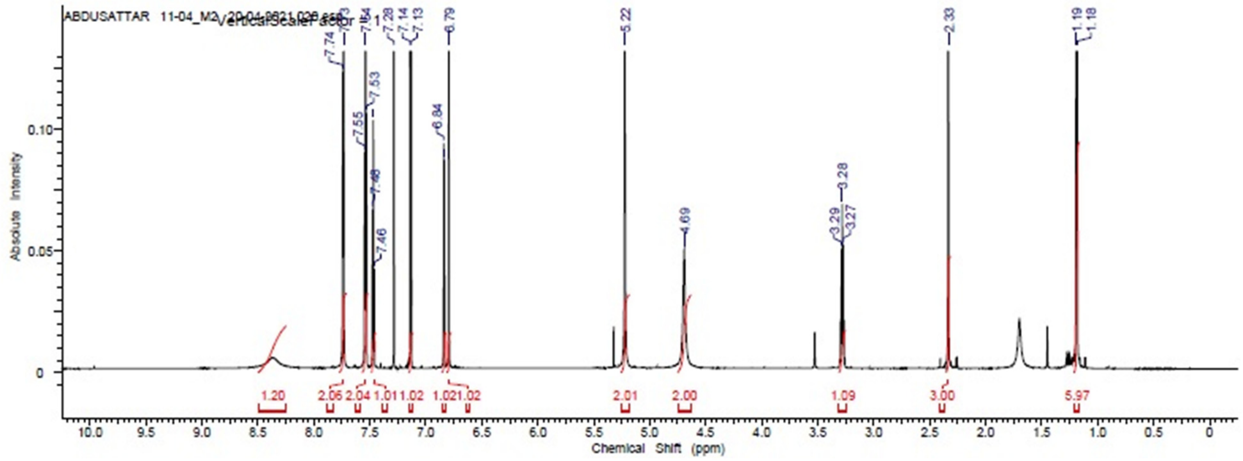

Compound 7

4/24/2021 9:31:50 PM

Acquisition Time (sec)	1.9268	*Comment*	Dr. Abdusattar Sampe 12-04 CDCL3			*Date*	20 Apr 2021 16:56:16
Date Stamp	20 Apr 2021 16:56:16						
File Name	D:\NMR Thymol oxadiazole triazole\Azizah_20210422\ABDUSATTAR_12-04_M3_20-04-2021\30\fid					*Frequency (MHz)*	850.15
Nucleus	1H	*Number of Transients*	32	*Origin*	spect	*Original Points Count*	32768
Owner	nmr	*Points Count*	32768	*Pulse Sequence*	zg30	*Receiver Gain*	12.46
SW(cyclical) (Hz)	17006.80	*Solvent*	CHLOROFORM-d			*Spectrum Offset (Hz)*	5250.0283
Spectrum Type	STANDARD	*Sweep Width (Hz)*	17006.28	*Temperature (degree C)*	25.000		

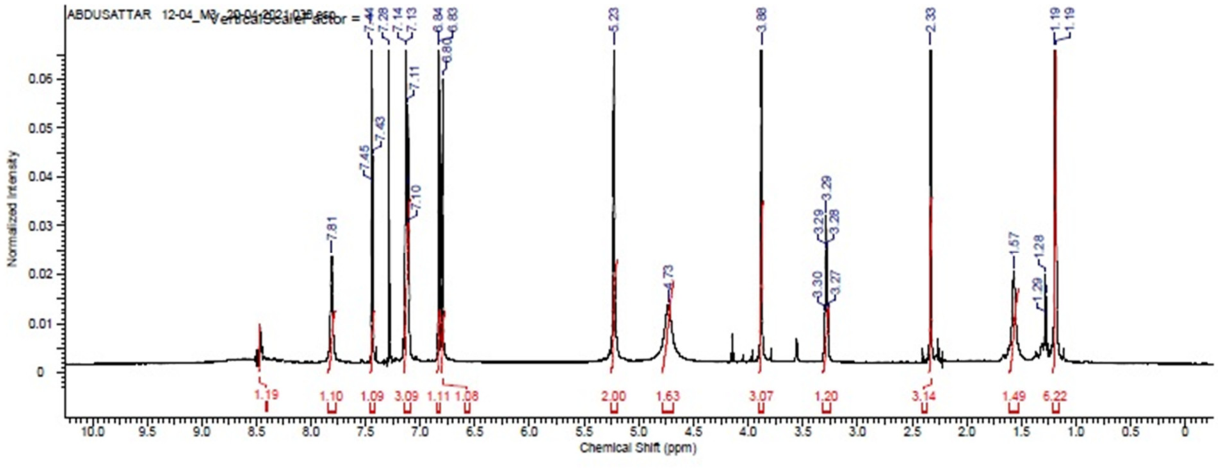

Compound 8

4/24/2021 9:45:03 PM

Acquisition Time (sec)	1.9268	*Comment*	Dr. Abdusattar Sampe 13-04 CDCL3			*Date*	20 Apr 2021 17:26:08
Date Stamp	20 Apr 2021 17:26:08						
File Name	D:\NMR Thymol oxadiazole triazole\Azizah 20210422\ABDUSATTAR 13-04 M4 20-04-2021\40\fid					*Frequency (MHz)*	850.15
Nucleus	1H	*Number of Transients*	32	*Origin*	spect	*Original Points Count*	32768
Owner	nmr	*Points Count*	32768	*Pulse Sequence*	zg30	*Receiver Gain*	12.46
SW(cyclical) (Hz)	17006.80	*Solvent*	CHLOROFORM-d			*Spectrum Offset (Hz)*	5250.0283
Spectrum Type	STANDARD	*Sweep Width (Hz)*	17006.28	*Temperature (degree C)*	25.000		

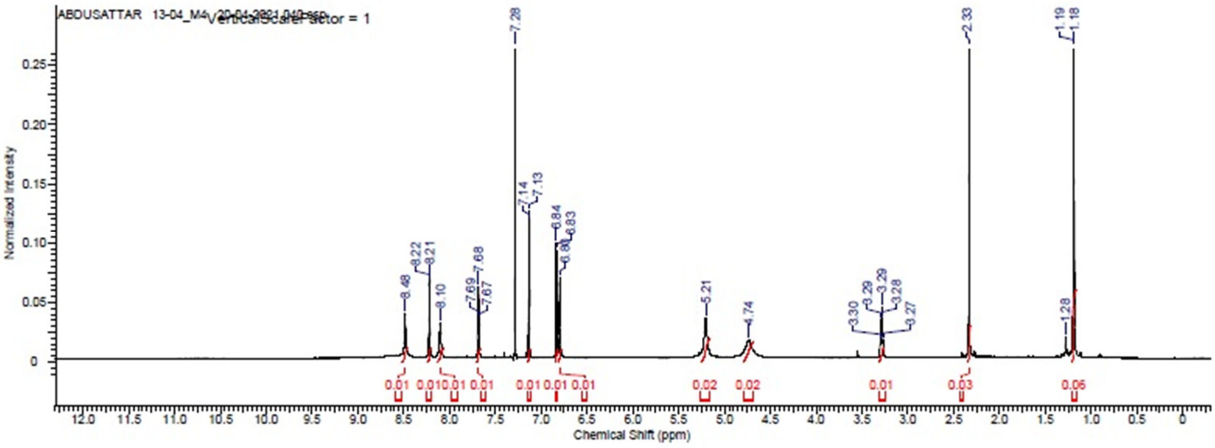

Compound 9

7/31/2021 2:14:02 PM

Acquisition Time (sec)	1.9268	*Comment*	Dr. Abdusattar Sampe 14-04 CDCL3			*Date*	20 Apr 2021 18:17:20
Date Stamp	20 Apr 2021 18:17:20			*File Name*	C:\Users\hp\Desktop\Azizah 20210422\ABDUSATTAR 14-04 M5 20-04-2021\50\fid		
Frequency (MHz)	850.15	*Nucleus*	1H	*Number of Transients*	32	*Origin*	spect
Original Points Count	32768	*Owner*	nmr	*Points Count*	32768	*Pulse Sequence*	zg30
Receiver Gain	12.46	*SW(cyclical) (Hz)*	17006.80	*Solvent*	CHLOROFORM-d		
Spectrum Offset (Hz)	5250.0283	*Spectrum Type*	STANDARD	*Sweep Width (Hz)*	17006.28	*Temperature (degree C)*	24.999

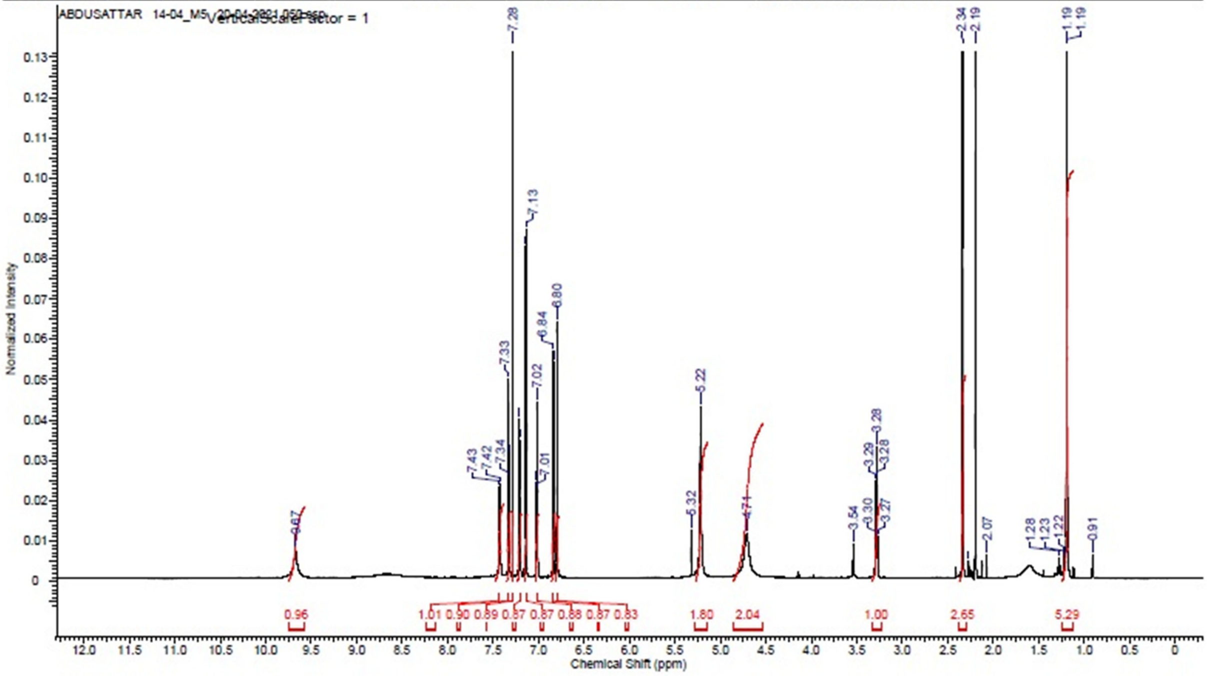

Compound 11

4/24/2021 10:56:08 PM

Acquisition Time (sec)	1.9268	*Comment*	Dr. Abdusattar Sampe 16-04 CDCL3			*Date*	20 Apr 2021 20:04:00
Date Stamp	20 Apr 2021 20:04:00						
File Name	D:\NMR Thymol oxadiazole triazole\Azizah_20210422\ABDUSATTAR_16-04_M7_20-04-2021\70\fid					*Frequency (MHz)*	850.15
Nucleus	1H	*Number of Transients*	32	*Origin*	spect	*Original Points Count*	32768
Owner	nmr	*Points Count*	32768	*Pulse Sequence*	zg30	*Receiver Gain*	12.46
SW(cyclical) (Hz)	17006.80	*Solvent*	CHLOROFORM-d			*Spectrum Offset (Hz)*	5250.0283
Spectrum Type	STANDARD	*Sweep Width (Hz)*	17006.28	*Temperature (degree C)*	25.000		

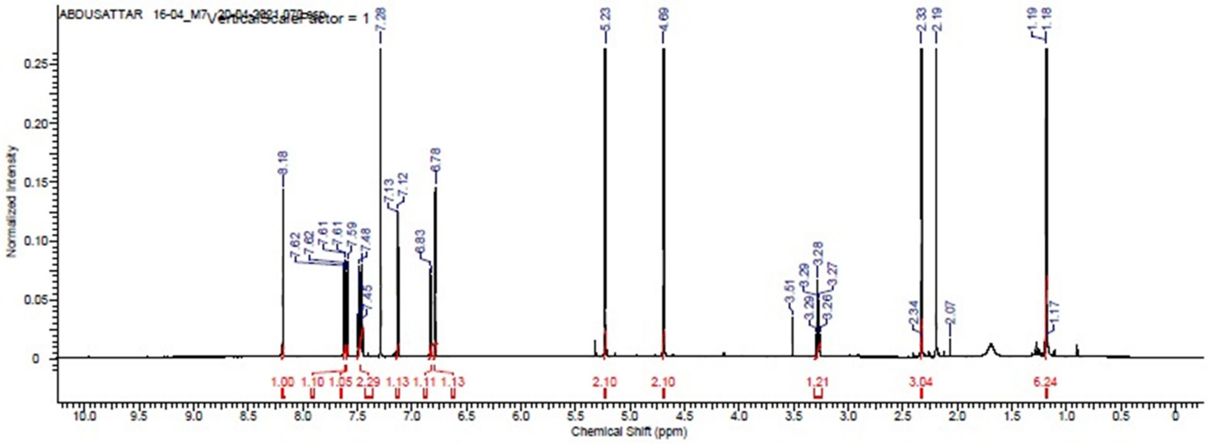

Compound 13

4/24/2021 11:13:54 PM

Acquisition Time (sec)	1.9268	*Comment*	Dr. Abdusattar Sampe 18-04 CDCL3			*Date*	20 Apr 2021 21:12:16
Date Stamp	20 Apr 2021 21:12:16						
File Name	D:\NMR Thymol oxadiazole triazole\Azizah_20210422\ABDUSATTAR_18-04_M9_20-04-2021\90\fid					*Frequency (MHz)*	850.15
Nucleus	1H	*Number of Transients*	32	*Origin*	spect	*Original Points Count*	32768
Owner	nmr	*Points Count*	32768	*Pulse Sequence*	zg30	*Receiver Gain*	12.46
SW(cyclical) (Hz)	17006.80	*Solvent*	CHLOROFORM-d			*Spectrum Offset (Hz)*	5250.0283
Spectrum Type	STANDARD	*Sweep Width (Hz)*	17006.28	*Temperature (degree C)*	25.000		

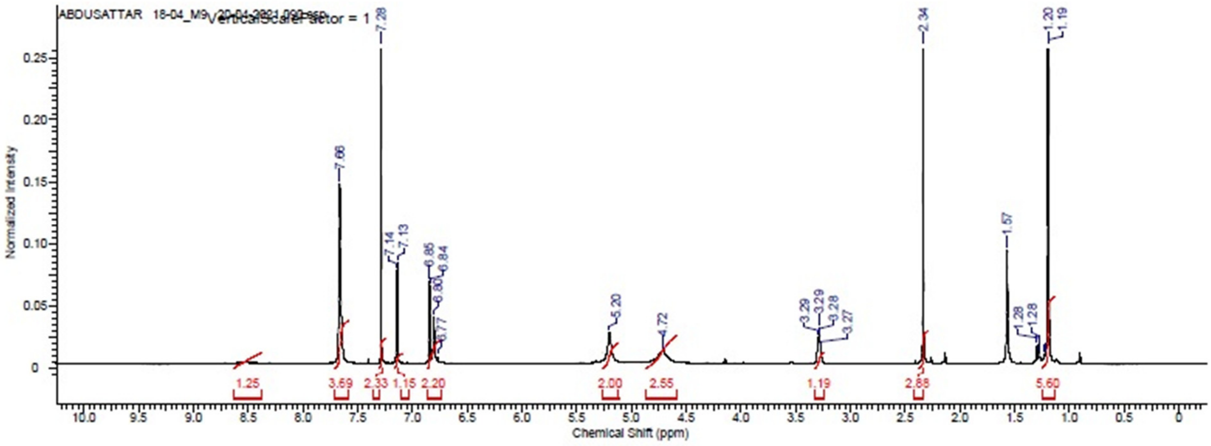

Compound 14

4/25/2021 12:35:16 AM

Acquisition Time (sec)	1.9268	*Comment*	Dr. Abdusattar Sampe 19-04 CDCL3			*Date*	20 Apr 2021 22:05:36
Date Stamp	20 Apr 2021 22:05:36						
File Name	D:\NMR Thymol oxadiazole triazole\Azizah_20210422\ABDUSATTAR_19-04_M10_20-04-2021\100\fid					*Frequency (MHz)*	850.15
Nucleus	1H	*Number of Transients*	32	*Origin*	spect	*Original Points Count*	32768
Owner	nmr	*Points Count*	32768	*Pulse Sequence*	zg30	*Receiver Gain*	12.46
SW(cyclical) (Hz)	17006.80	*Solvent*	CHLOROFORM-d			*Spectrum Offset (Hz)*	5250.0283
Spectrum Type	STANDARD	*Sweep Width (Hz)*	17006.28	*Temperature (degree C)*	24.998		

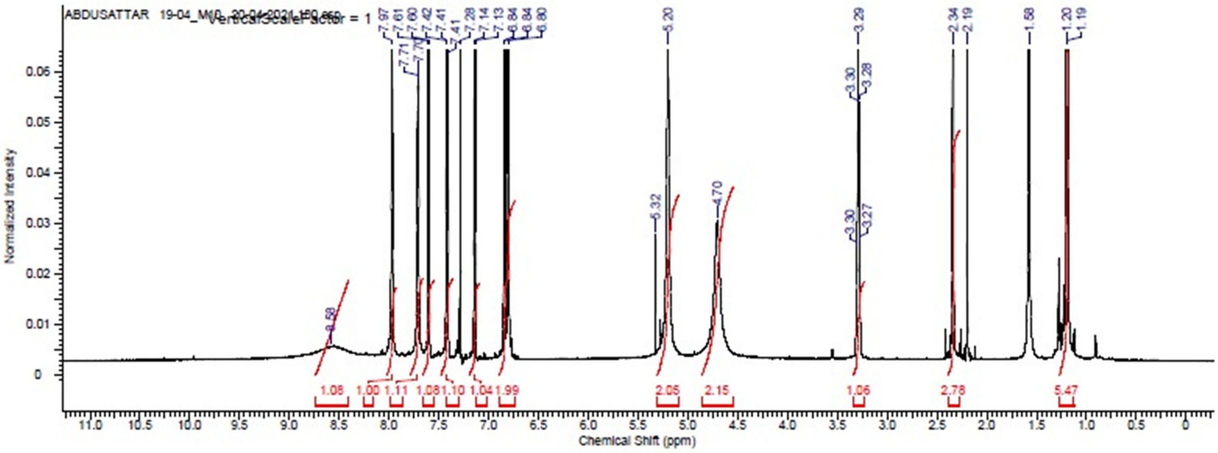

Compound 15

4/25/2021 12:42:29 AM

Acquisition Time (sec)	1.9268	*Comment*	Dr. Abdusattar Sampe 20-04 CDCL3			*Date*	20 Apr 2021 22:58:56
Date Stamp	20 Apr 2021 22:58:56						
File Name	D:\NMR Thymol oxadiazole triazole\Azizah_20210422\ABDUSATTAR_20-04_M11_20-04-2021\110\fid					*Frequency (MHz)*	850.15
Nucleus	1H	*Number of Transients*	32	*Origin*	spect	*Original Points Count*	32768
Owner	nmr	*Points Count*	32768	*Pulse Sequence*	zg30	*Receiver Gain*	12.46
SW(cyclical) (Hz)	17006.80	*Solvent*	CHLOROFORM-d			*Spectrum Offset (Hz)*	5250.0283
Spectrum Type	STANDARD	*Sweep Width (Hz)*	17006.28	*Temperature (degree C)*	25.000		

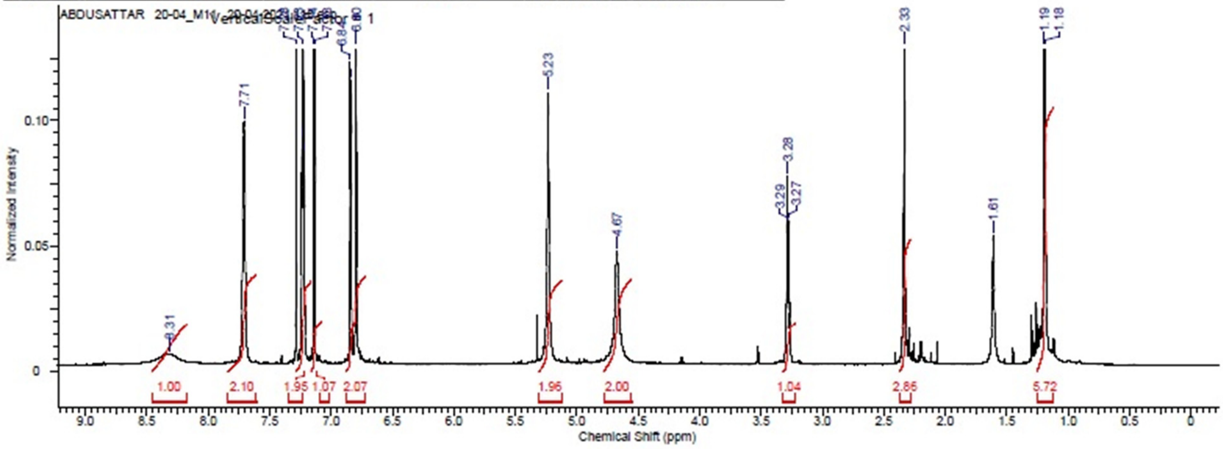

Compound 16

4/25/2021 12:48:47 AM

Acquisition Time (sec)	1.9268	*Comment*	Dr. Abdusattar Sampe 21-04 CDCL3			*Date*	20 Apr 2021 23:26:40
Date Stamp	20 Apr 2021 23:26:40						
File Name	D:\NMR Thymol oxadiazole triazole\Azizah_20210422\ABDUSATTAR_21-04_M12_20-04-2021\120\fid					*Frequency (MHz)*	850.15
Nucleus	1H	*Number of Transients*	32	*Origin*	spect	*Original Points Count*	32768
Owner	nmr	*Points Count*	32768	*Pulse Sequence*	zg30	*Receiver Gain*	12.46
SW(cyclical) (Hz)	17006.80	*Solvent*	CHLOROFORM-d			*Spectrum Offset (Hz)*	5250.0283
Spectrum Type	STANDARD	*Sweep Width (Hz)*	17006.28	*Temperature (degree C)*	25.000		

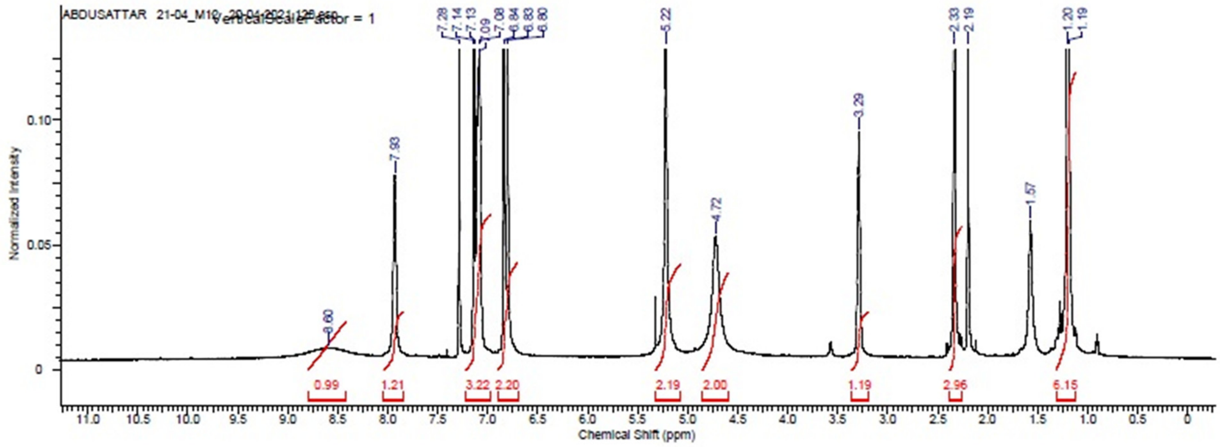

Figure S10-S18: ^{13}C NMR of final compounds

Compound 6

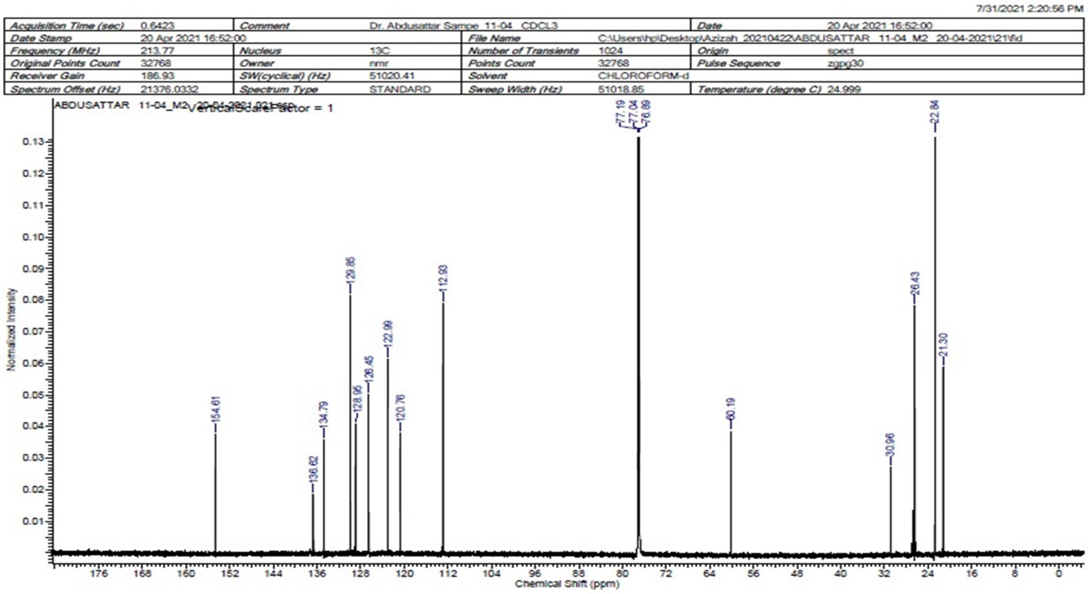

Compound 7

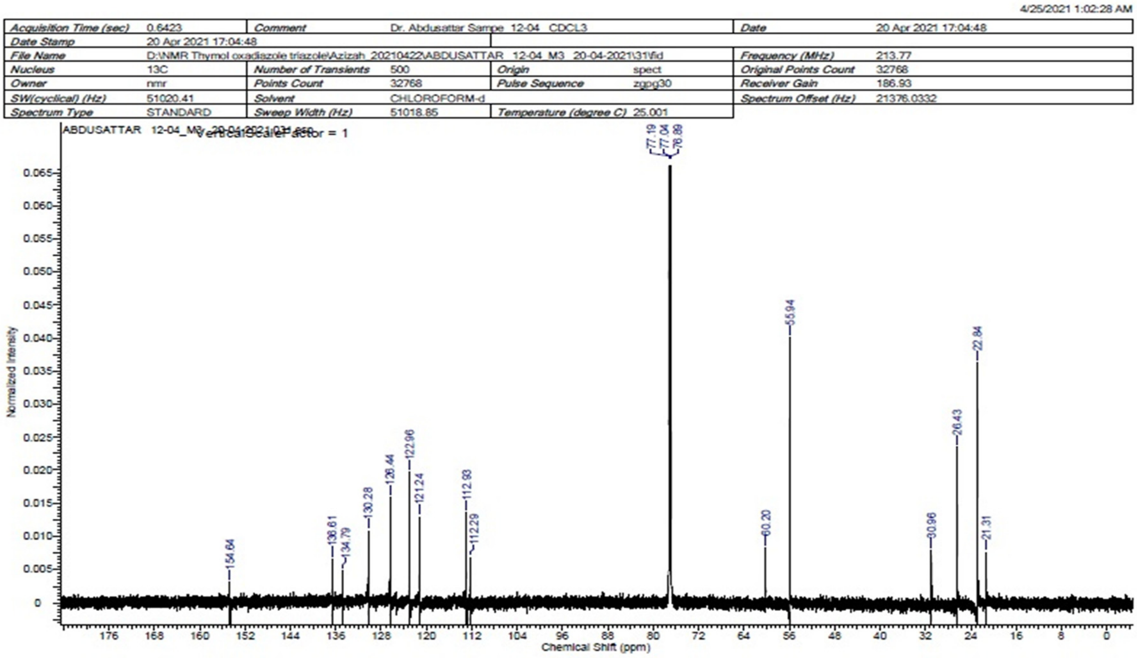

Compound 8

4/25/2021 1:20:18 AM

Acquisition Time (sec)	0.6423	*Comment*	Dr. Abdusattar Sampe 13-04 CDCL3			*Date*	20 Apr 2021 18:13:04
Date Stamp	20 Apr 2021 18:13:04						
File Name	D:\NMR Thymol oxadiazole triazole\Azizah 20210422\ABDUSATTAR 13-04 M4 20-04-2021\41\fid					*Frequency (MHz)*	213.77
Nucleus	13C	*Number of Transients*	1024	*Origin*	spect	*Original Points Count*	32768
Owner	nmr	*Points Count*	32768	*Pulse Sequence*	zgpg30	*Receiver Gain*	186.93
SW(cyclical) (Hz)	51020.41	*Solvent*	CHLOROFORM-d	*Spectrum Offset (Hz)*	21376.0332	*Spectrum Type*	STANDARD
Sweep Width (Hz)	51018.85	*Temperature (degree C)*	25.000				

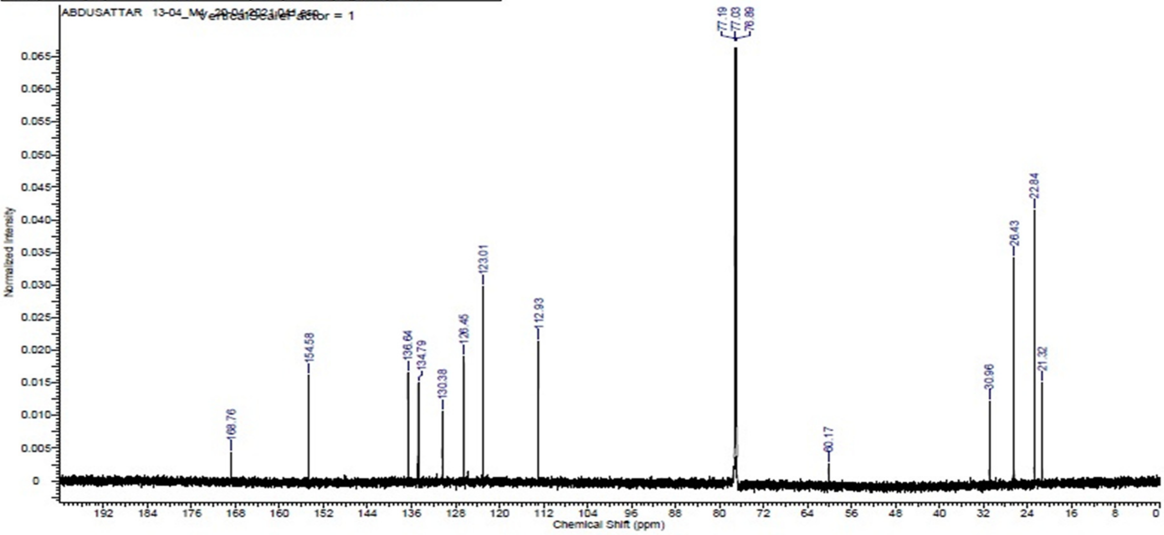

Compound 9

4/25/2021 1:23:18 AM

Acquisition Time (sec)	0.6423	*Comment*	Dr. Abdusattar Sampe 14-04 CDCL3			*Date*	20 Apr 2021 19:06:24
Date Stamp	20 Apr 2021 19:06:24						
File Name	D:\NMR Thymol oxadiazole triazole\Azizah 20210422\ABDUSATTAR 14-04 M5 20-04-2021\51\fid					*Frequency (MHz)*	213.77
Nucleus	13C	*Number of Transients*	1024	*Origin*	spect	*Original Points Count*	32768
Owner	nmr	*Points Count*	32768	*Pulse Sequence*	zgpg30	*Receiver Gain*	186.93
SW(cyclical) (Hz)	51020.41	*Solvent*	CHLOROFORM-d	*Spectrum Offset (Hz)*	21376.0332	*Spectrum Type*	STANDARD
Sweep Width (Hz)	51018.85	*Temperature (degree C)*	25.002				

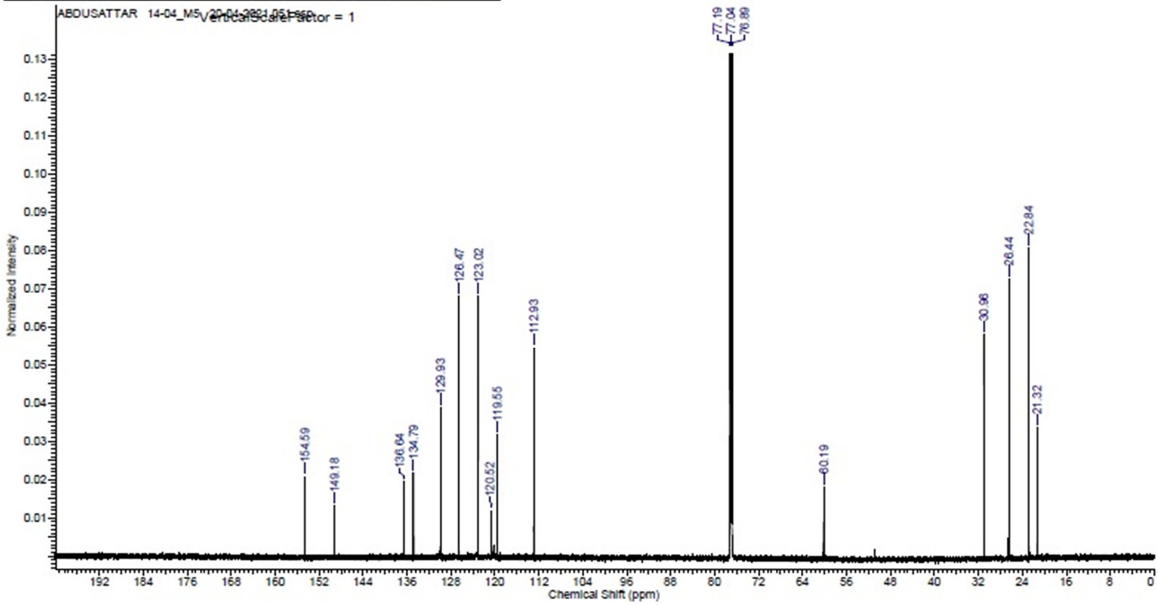

Compound 11

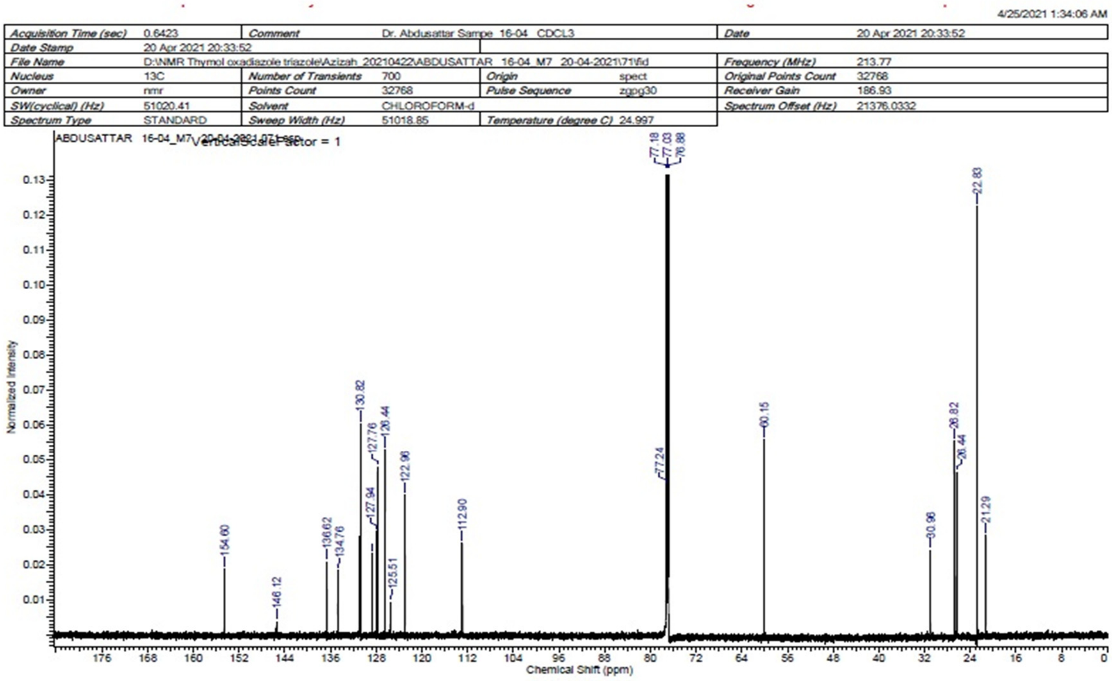

Compound 13

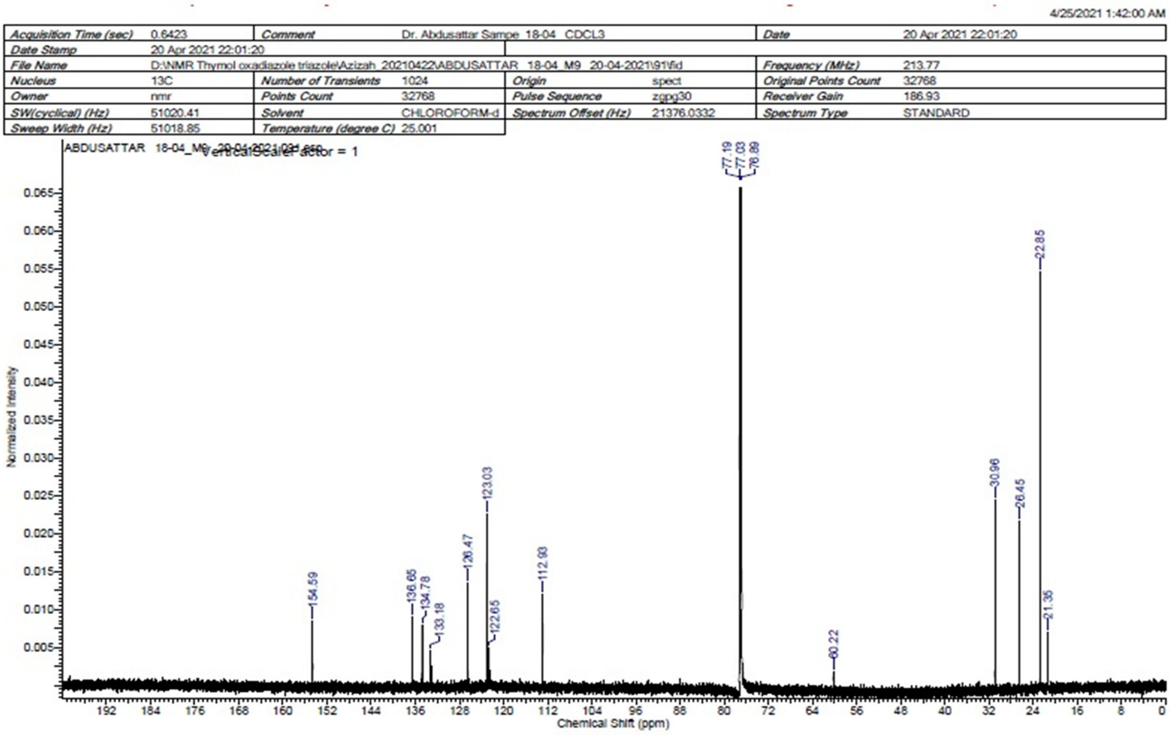

Compound 14

7/31/2021 3:53:41 PM

Acquisition Time (sec)	0.6423	*Comment*	Dr. Abdusattar Sampe 19-04 CDCL3			*Date*	20 Apr 2021 22:52:32
Date Stamp	20 Apr 2021 22:52:32			*File Name*	C:\Users\hp\Desktop\Azizah 20210422\ABDUSATTAR 19-04 M10 20-04-2021\101\fid		
Frequency (MHz)	213.77	*Nucleus*	13C	*Number of Transients*	1024	*Origin*	spect
Original Points Count	32768	*Owner*	nmr	*Points Count*	32768	*Pulse Sequence*	zgpg30
Receiver Gain	186.93	*SW(cyclical) (Hz)*	51020.41	*Solvent*	CHLOROFORM-d		
Spectrum Offset (Hz)	21376.0332	*Spectrum Type*	STANDARD	*Sweep Width (Hz)*	51018.85	*Temperature (degree C)*	25.001

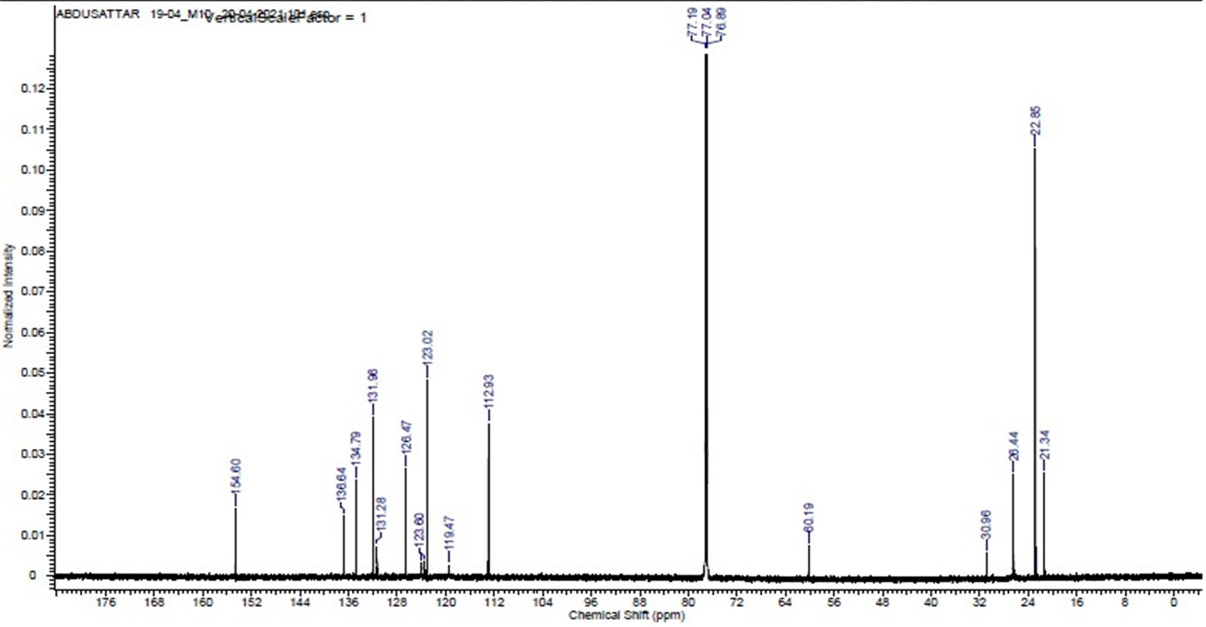

Compound 15

7/31/2021 3:55:32 PM

Acquisition Time (sec)	0.6423	*Comment*	Dr. Abdusattar Sampe 20-04 CDCL3			*Date*	20 Apr 2021 23:13:52
Date Stamp	20 Apr 2021 23:13:52			*File Name*	C:\Users\hp\Desktop\Azizah 20210422\ABDUSATTAR 20-04 M11 20-04-2021\111\fid		
Frequency (MHz)	213.77	*Nucleus*	13C	*Number of Transients*	500	*Origin*	spect
Original Points Count	32768	*Owner*	nmr	*Points Count*	32768	*Pulse Sequence*	zgpg30
Receiver Gain	186.93	*SW(cyclical) (Hz)*	51020.41	*Solvent*	CHLOROFORM-d		
Spectrum Offset (Hz)	21376.0332	*Spectrum Type*	STANDARD	*Sweep Width (Hz)*	51018.85	*Temperature (degree C)*	24.999

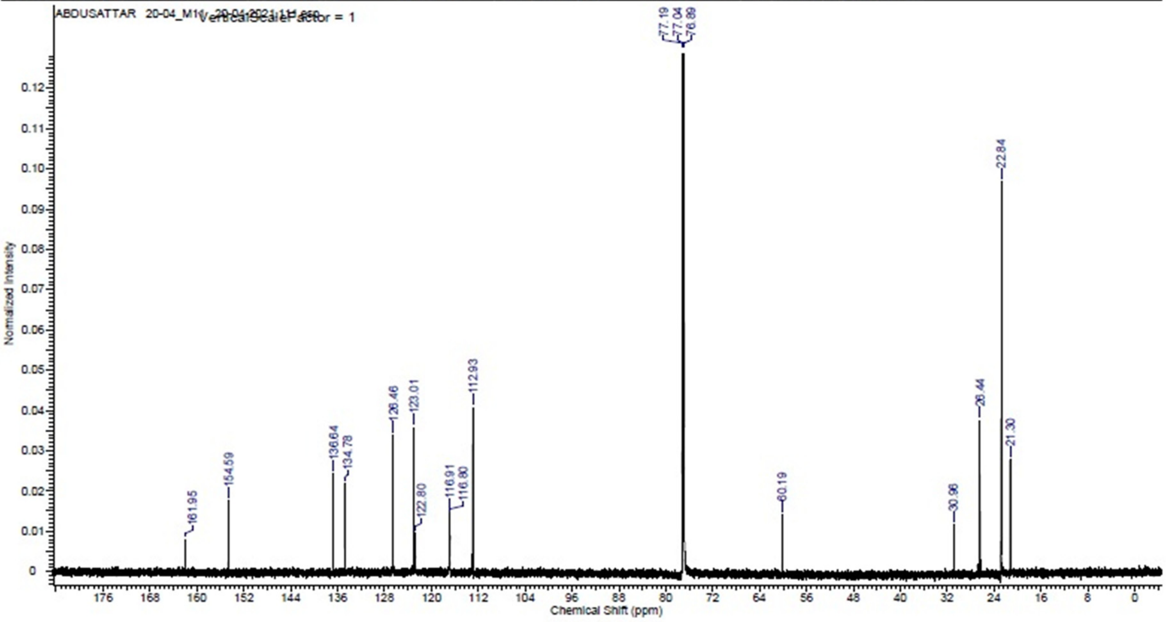

Compound 16

4/25/2021 1:52:43 AM

Acquisition Time (sec)	0.6423	*Comment*	Dr. Abdusattar Sampe 21-04 CDCL3			*Date*	21 Apr 2021 00:15:44
Date Stamp	21 Apr 2021 00:15:44						
File Name	D:\NMR Thymol oxadiazole triazole\Azizah 20210422\ABDUSATTAR 21-04 M12 20-04-2021\121\fid					*Frequency (MHz)*	213.77
Nucleus	13C	*Number of Transients*	1024	*Origin*	spect	*Original Points Count*	32768
Owner	nmr	*Points Count*	32768	*Pulse Sequence*	zgpg30	*Receiver Gain*	186.93
SW(cyclical) (Hz)	51020.41	*Solvent*	CHLOROFORM-d	*Spectrum Offset (Hz)*	21376.0332	*Spectrum Type*	STANDARD
Sweep Width (Hz)	51018.85	*Temperature (degree C)*	25.001				

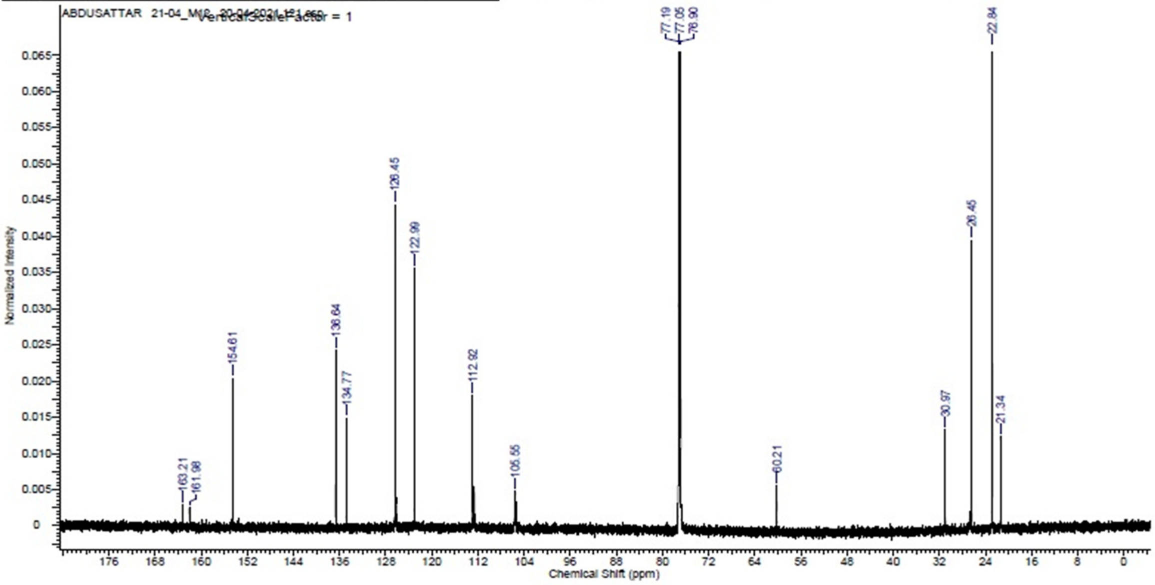

Figure S19-S29: Mass of final compounds

Compound 6

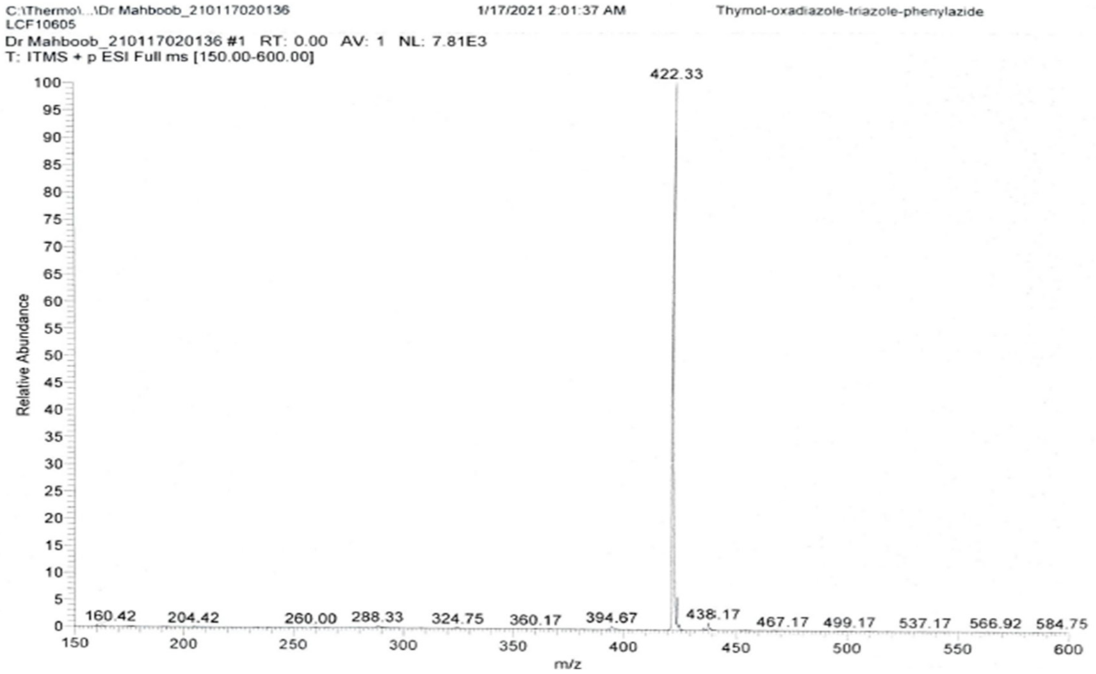

Compound 7

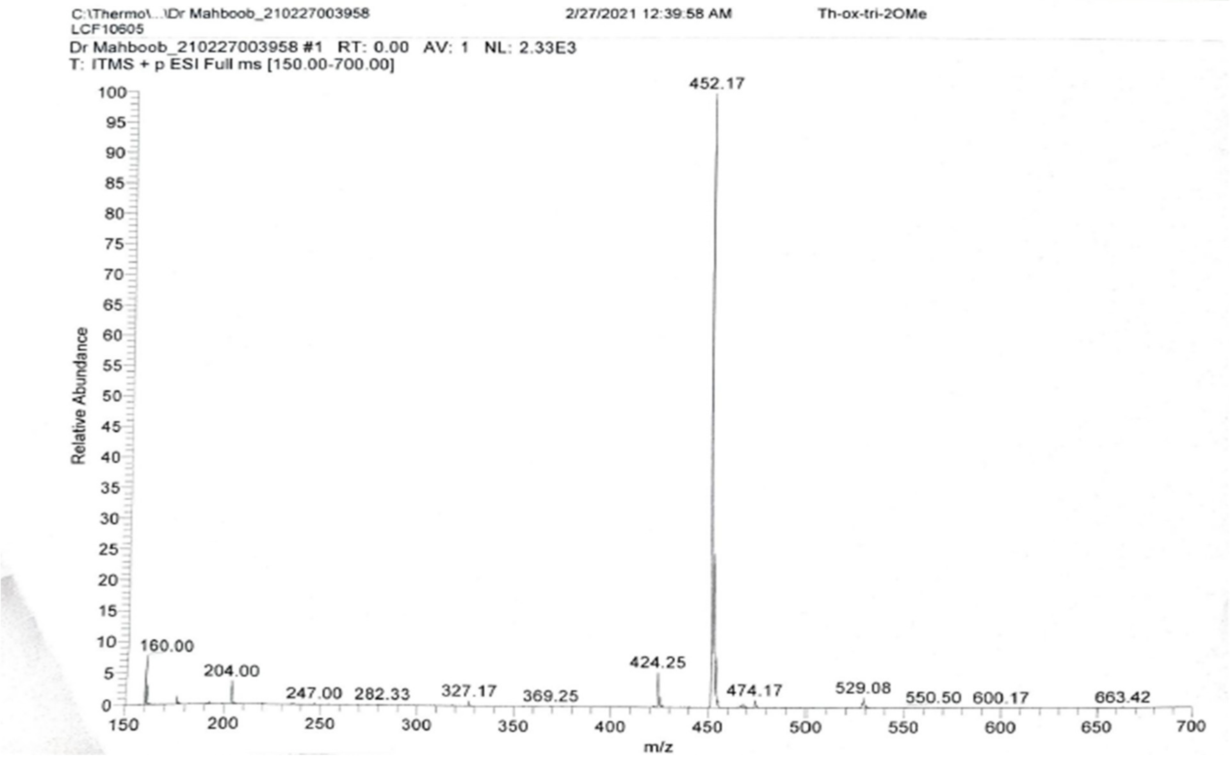

Compound 8

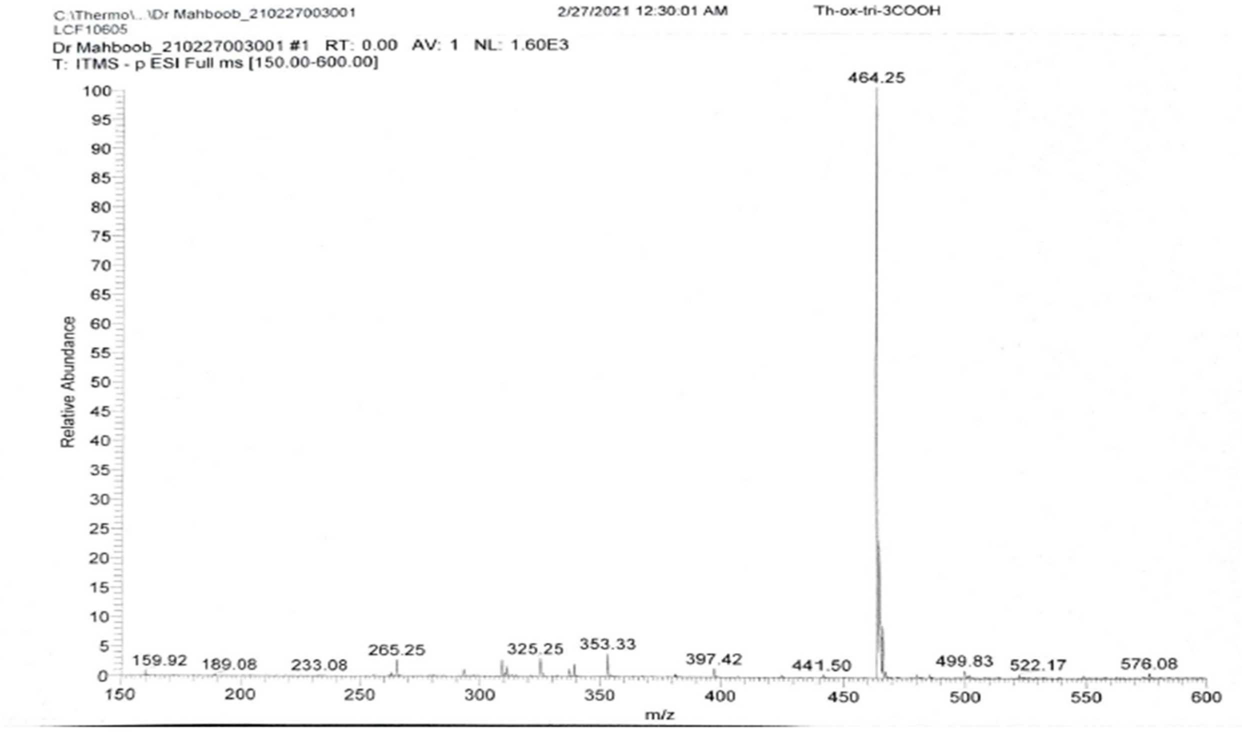

Compound 9

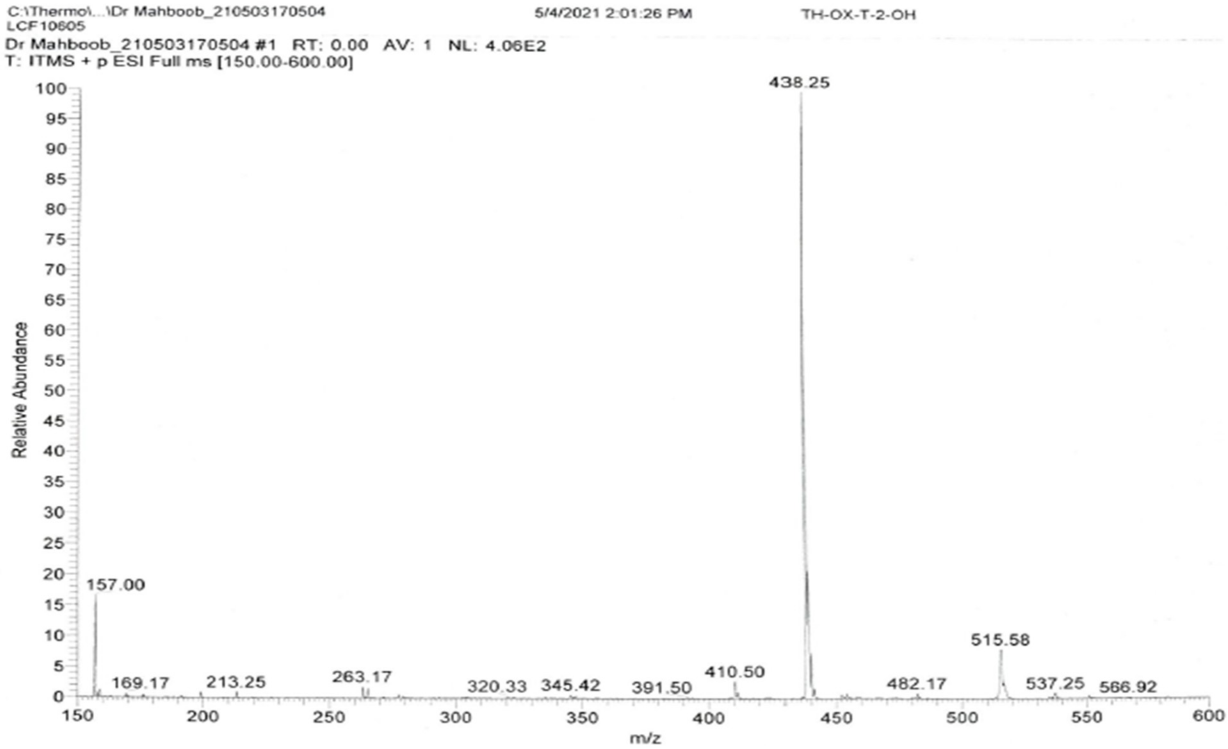

Compound 10

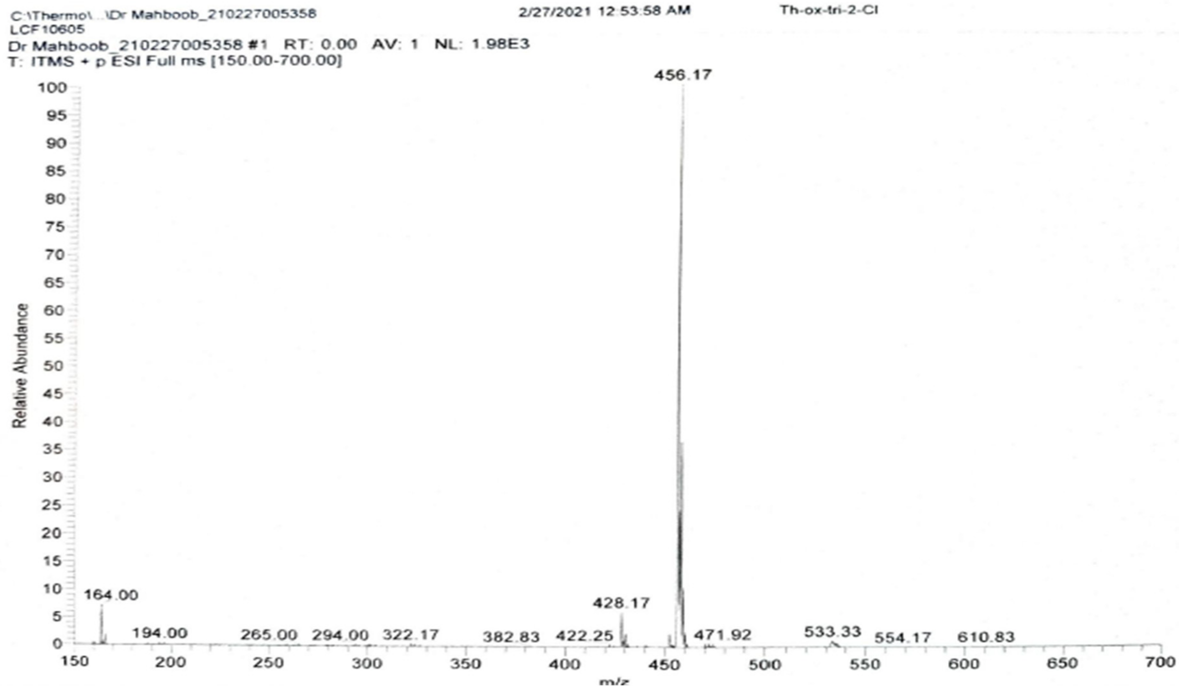

Compound 11

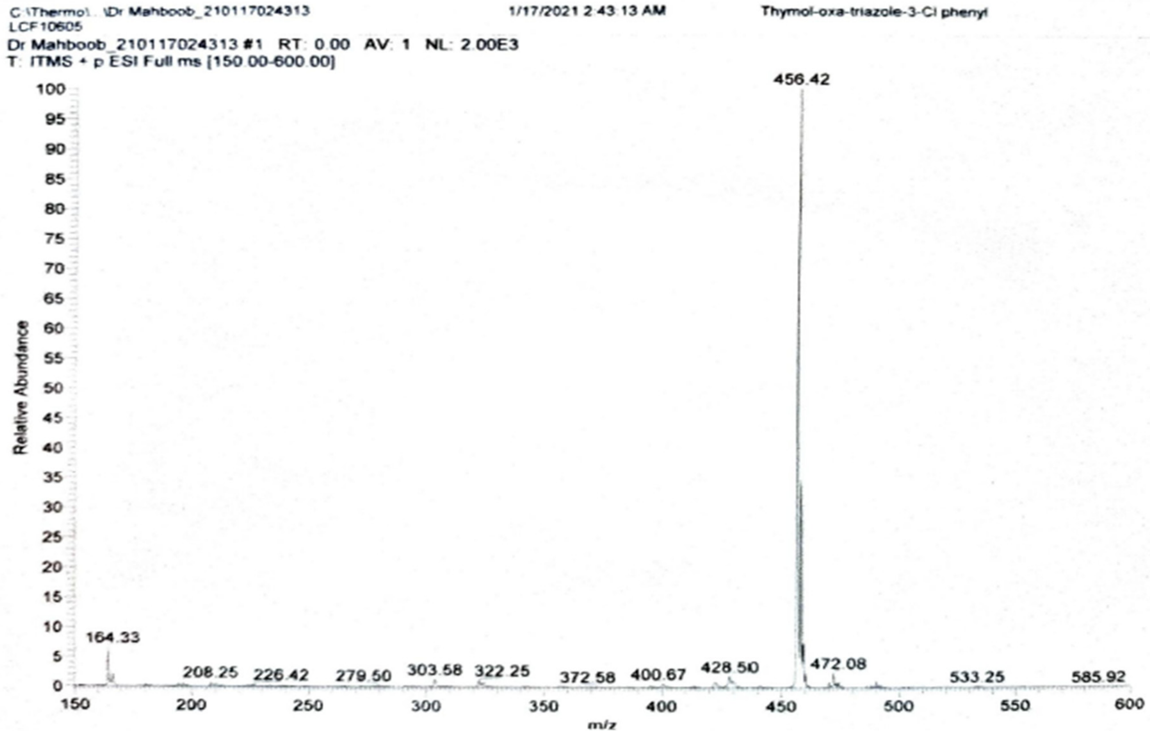

Compound 12

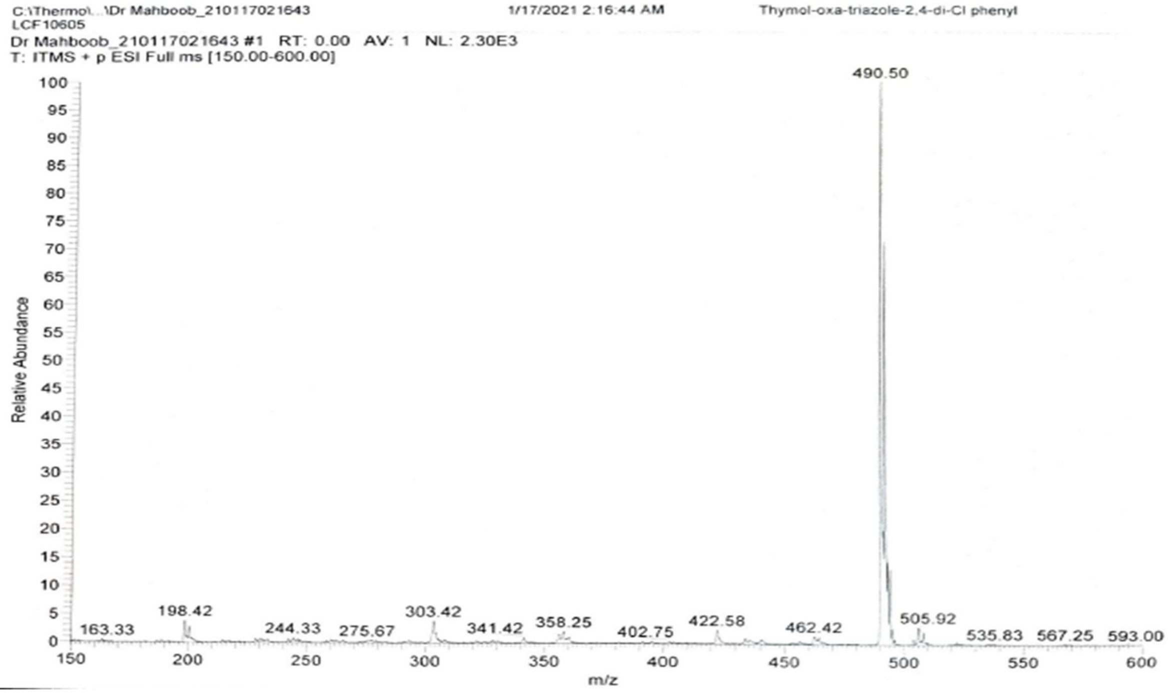

Compound 13

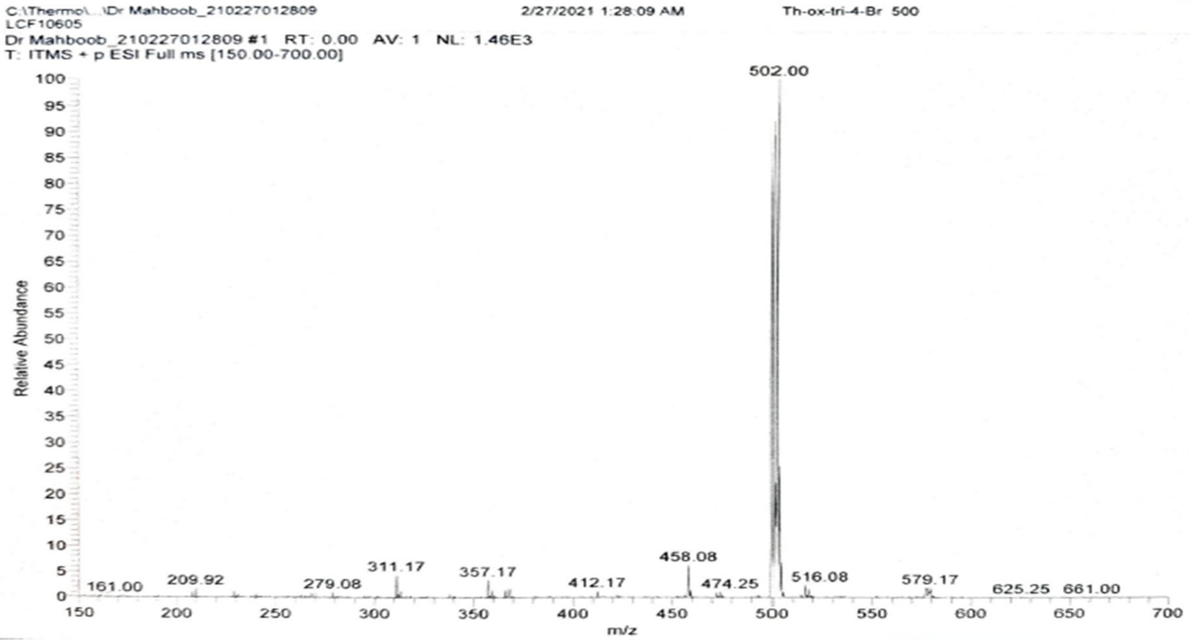

Compound 14

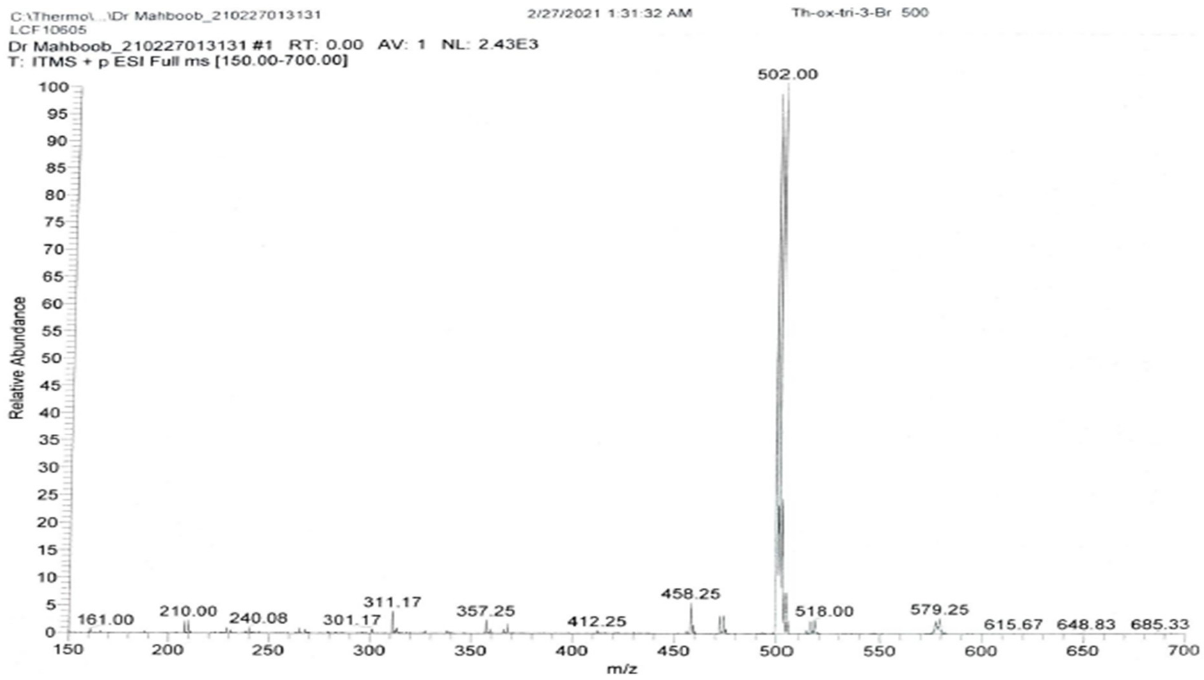

Compound 15

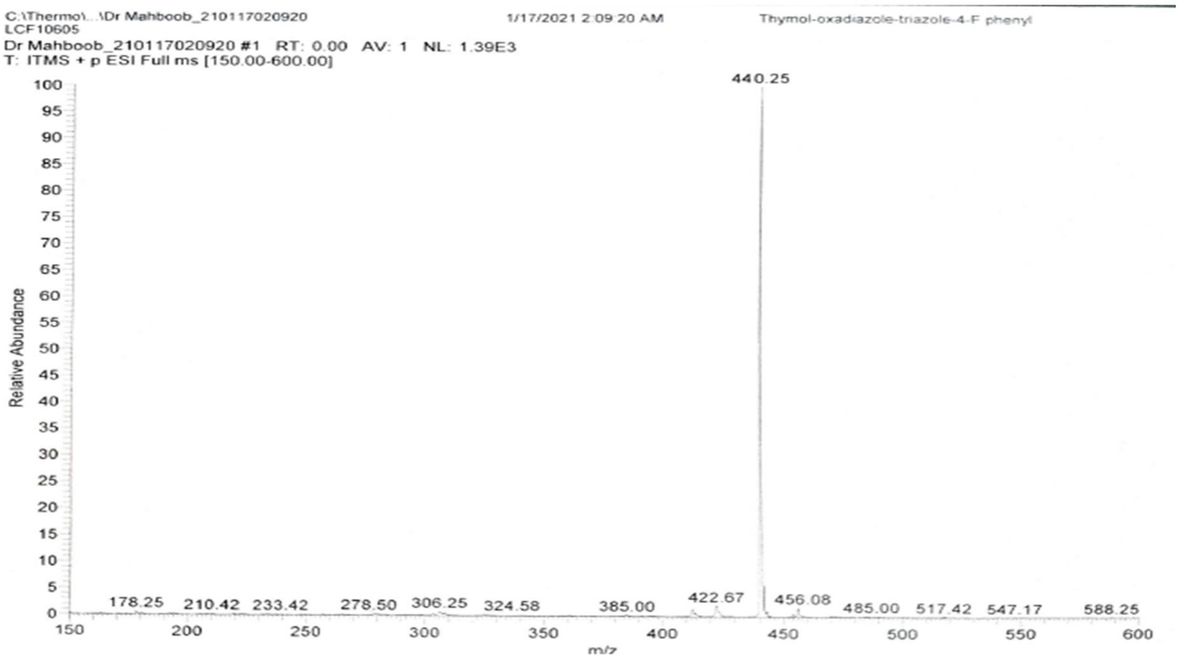

Compound 16

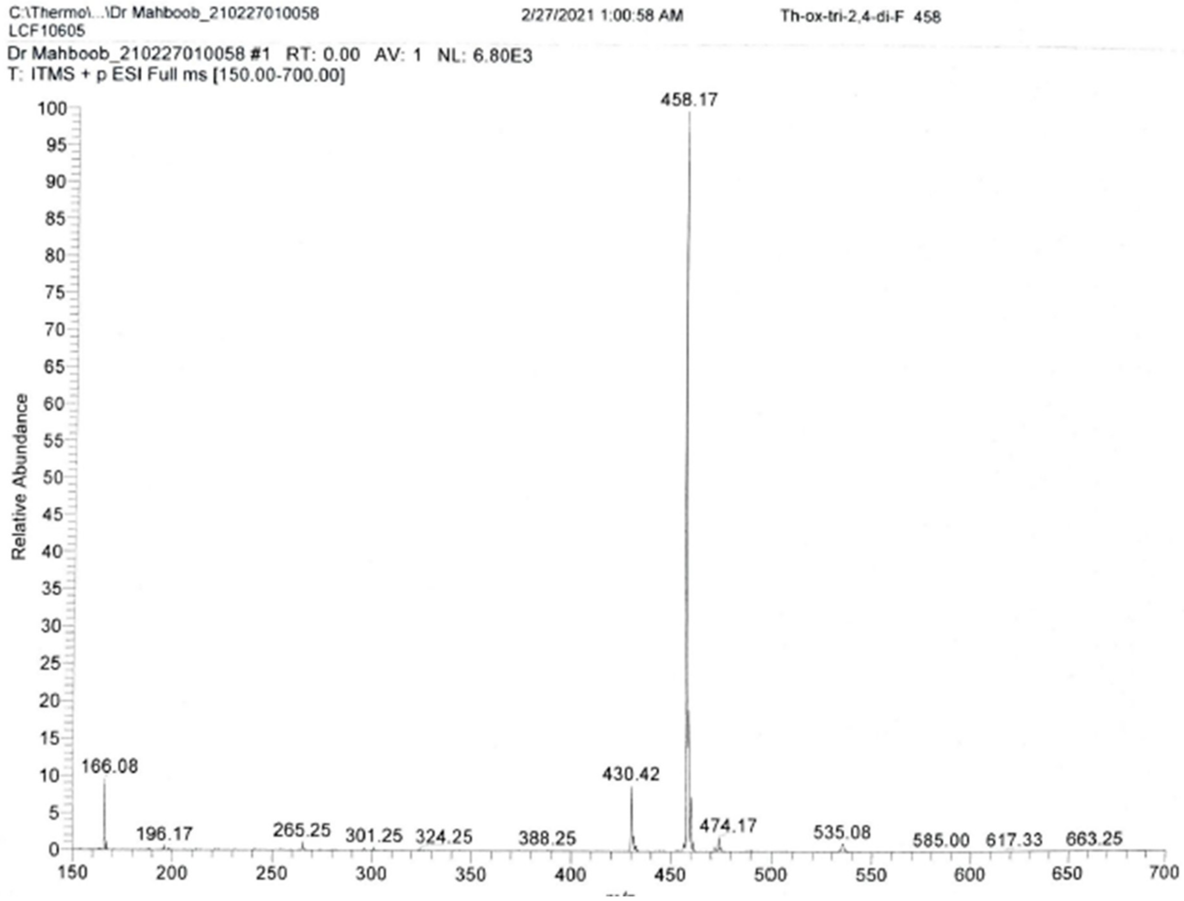
C:\Thermo\...\Dr Mahboob_210227010058
LCF10605
2/27/2021 1:00:58 AM
Th-ox-tri-2,4-di-F 458
Dr Mahboob_210227010058 #1 RT: 0.00 AV: 1 NL: 6.80E3
T: ITMS + p ESI Full ms [150.00-700.00]
Relative Abundance
458.17
166.08
196.17
265.25
301.25
324.25
388.25
430.42
474.17
535.08
585.00
617.33
663.25

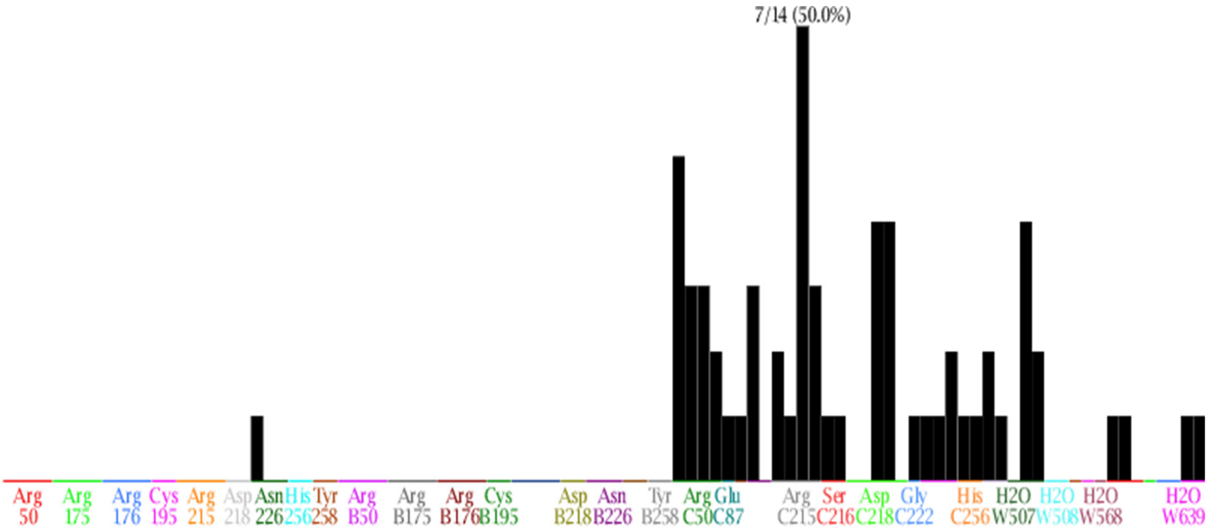

FigureS30. PLIF histogram which represented the interacted docked compounds with residues of thymidylate synthase.

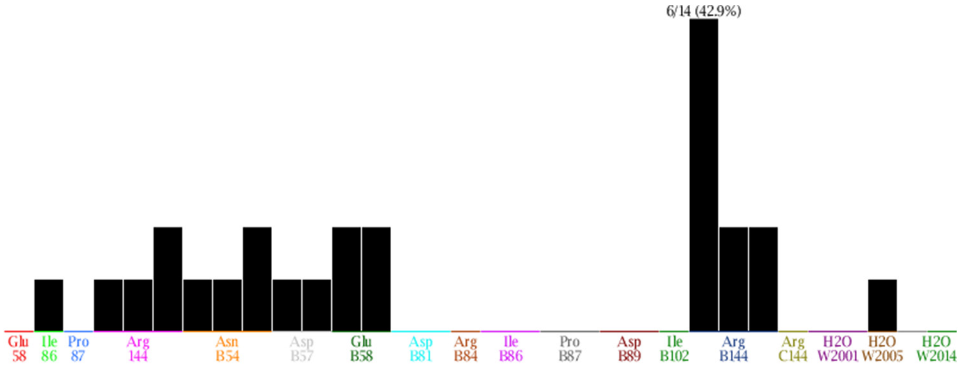

FigureS31. PLIF histogram which represented the interacted docked compounds with residues of DNA gyrase (4uro).
